# Toward Green Atom Transfer Radical Polymerization: Current Status and Future Challenges

**DOI:** 10.1002/advs.202106076

**Published:** 2022-02-17

**Authors:** Sylwia Dworakowska, Francesca Lorandi, Adam Gorczyński, Krzysztof Matyjaszewski

**Affiliations:** ^1^ Department of Chemistry Carnegie Mellon University 4400 Fifth Avenue Pittsburgh PA 15213 USA; ^2^ Faculty of Chemical Engineering and Technology Cracow University of Technology Warszawska 24 Cracow 31‐155 Poland; ^3^ Department of Industrial Engineering University of Padova via Marzolo 9 Padova 35131 Italy; ^4^ Faculty of Chemistry Adam Mickiewicz University Uniwersytetu Poznańskiego 8 Poznań 61‐614 Poland

**Keywords:** atom transfer radical polymerization, biobased polymers, degradable polymers, depolymerization, green chemistry

## Abstract

Reversible‐deactivation radical polymerizations (RDRPs) have revolutionized synthetic polymer chemistry. Nowadays, RDRPs facilitate design and preparation of materials with controlled architecture, composition, and functionality. Atom transfer radical polymerization (ATRP) has evolved beyond traditional polymer field, enabling synthesis of organic–inorganic hybrids, bioconjugates, advanced polymers for electronics, energy, and environmentally relevant polymeric materials for broad applications in various fields. This review focuses on the relation between ATRP technology and the 12 principles of green chemistry, which are paramount guidelines in sustainable research and implementation. The green features of ATRP are presented, discussing the environmental and/or health issues and the challenges that remain to be overcome. Key discoveries and recent developments in green ATRP are highlighted, while providing a perspective for future opportunities in this area.

## Graphical abstract

Atom transfer radical polymerization (ATRP) is a powerful technique for the controlled synthesis of functional polymeric materials. Recent advances in the implementation of the twelve principles of green chemistry in ATRP are presented, highlighting the remaining challenges and opportunities for ATRP to play a fundamental role in the transition toward sustainable polymer production.

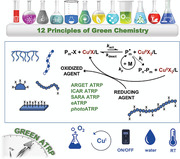

## 1 Introduction

Polymers are ubiquitous in our lives. Clothes, shoes, electronic devices, medical equipment, pharmaceuticals, domestic appliances all have polymeric components, as have cars, airplanes, other transportation vehicles or even concrete and pavements. Polymeric materials are characterized by exceptional durability, functionality, and low cost. Polymers considerably improve our wellness: healthy drinking water is enabled by purification through polymeric membranes, food is stored in plastic packaging that prevents external contamination, and polymer coatings favor the oral administration of drugs. The rising number of infectious disease outbreaks in the last few decades, especially the 2019 coronavirus outbreak, exacerbated the demand for facial masks and respirators, which are made of polymers.

During the XIX century, Rayon and vulcanized rubber were produced by modification of natural polymers, while the first synthetic polymer, phenol‐formaldehyde resin (Bakelite), was invented in 1907 and since then, global plastic production raised exponentially.^[^ ^1^ ^]^ Large production of synthetic polymers began during the World War II and has driven such a rapid growth of polymeric materials that the second half of the XX century is often termed the “plastic age.”^[^ ^2^ ^]^ In practice, 360 million metric tons (Mt) of synthetic polymers were produced worldwide in 2018, and the polymer industry employs 1.8 million people in the United States and over 1.6 million in Europe.^[^ ^3^ ^]^ Moreover, it is noteworthy that in 2020, polymer science celebrated its 100th birthday, a century after the milestone publication “On polymerization” by Hermann Staudinger.^[^ ^4^ ^]^

Natural polymers such as polysaccharides and polypeptides were known since ancient human history and processed to make papers and clothing. However, nowadays more than 90% of synthetic polymers are derived from fossil feedstocks.^[^ ^1^ ^]^ Plastic production accounts for *ca*. 6% of global oil consumption. This number is projected to increase by 20% until 2050, if the market will keep growing at the current rate with no change in feedstock composition.^[^ ^5^ ^]^ Some estimates show that 6300 Mt of plastic waste were generated worldwide between 1950 and 2015, of which *ca*. 9% was recycled (typically only once), 12% was incinerated to generate energy, while the remaining was accumulated in landfills or dumped into the environment.^[^ ^6^ ^]^ At this pace, it is expected that more plastics than fish will populate the oceans by 2050.^[^ ^1^ ^]^ Polymer scientists are at the forefront of reversing this scenario.

The incineration of plastic waste to produce energy enables to treat complex mixtures, however it emits CO_2_ and toxic byproducts, and recovers much less energy than the amount that can be saved by recycling.^[^ ^5^ ^]^ On the other hand, mechanical recycling of plastic waste causes deterioration of some material properties, due to chain scission or cross‐linking reactions induced by acids, heat, or contamination with food or other polymers.^[^ ^2^ ^]^ Conversely, chemical recycling targets the conversion of polymers into small molecules, such as monomers (i.e., depolymerization) or other chemical feedstocks, of sufficiently good quality to be reprocessed, replacing fossil feedstocks.^[^ ^7^ ^]^ Catalysts are often necessary to reduce the temperature and increase the selectivity of chemical recycling processes. Depolymerization can be triggered by heating polymers above the ceiling temperature (*T*_c_, i.e., the temperature at which the rates of polymerization and depolymerization are equal)^[^ ^3a^ ^]^ to recover high‐purity monomers, however at high‐temperature side reactions hamper the selectivity. Alternatively, polyesters and polyamides can be degraded via hydrolysis of ester or amide linkages, respectively. This opens up the perspective of designing polymers with degradation in mind, by incorporating functionalities that can be degraded upon use but that preserve the desired mechanical properties during life.^[^ ^8^ ^]^ However, not only thermoplastics, but also thermosets can be designed as reprocessable and recyclable materials, by introducing reversible cross‐links through weak covalent bonds, Diels–Alder cycloadducts, supramolecular chemistries, or associative exchange reactions.^[^ ^3a^ ^]^ One of the major challenges in the design of degradable polymers is considering the effects of a complex environment, such as a landfill, on the actual degradation rate and pathways.^[^ ^2^ ^]^ Introducing stimuli‐responsive functionalities partially addresses this challenge, leading to smart polymers with triggerable degradation.^[^ ^2^, ^3^ ^]^ Other promising strategies to overcome the need for separating waste are designing polymer compatibilizers and upcycling to create high‐value materials.^[^ ^5^ ^]^

Besides strategies focused on end‐of‐life and reuse, advancing the green birth of polymeric materials is equally important. In particular, fossil feedstocks can be replaced by bioderived monomers.^[^ ^2^, ^9^ ^]^ One of the major challenges in this context is to develop processes cost competitive with the traditional petrochemical industry. Alongside, it is fundamental to control the morphology and architecture of bioderived polymers, and to understand structure–property relationships to impart desired thermal and mechanical behaviors. In parallel, it is imperative to enhance the green character of production processes. Energy‐efficient and atom‐economical syntheses can be accessed by using catalysts and/or waterborne systems. External stimuli such as light or electricity give access to milder reaction conditions while taking advantage of renewable energy sources. Flow chemistry technologies can replace batch processes achieving continuous and rapid product supply.

**Scheme 1:**
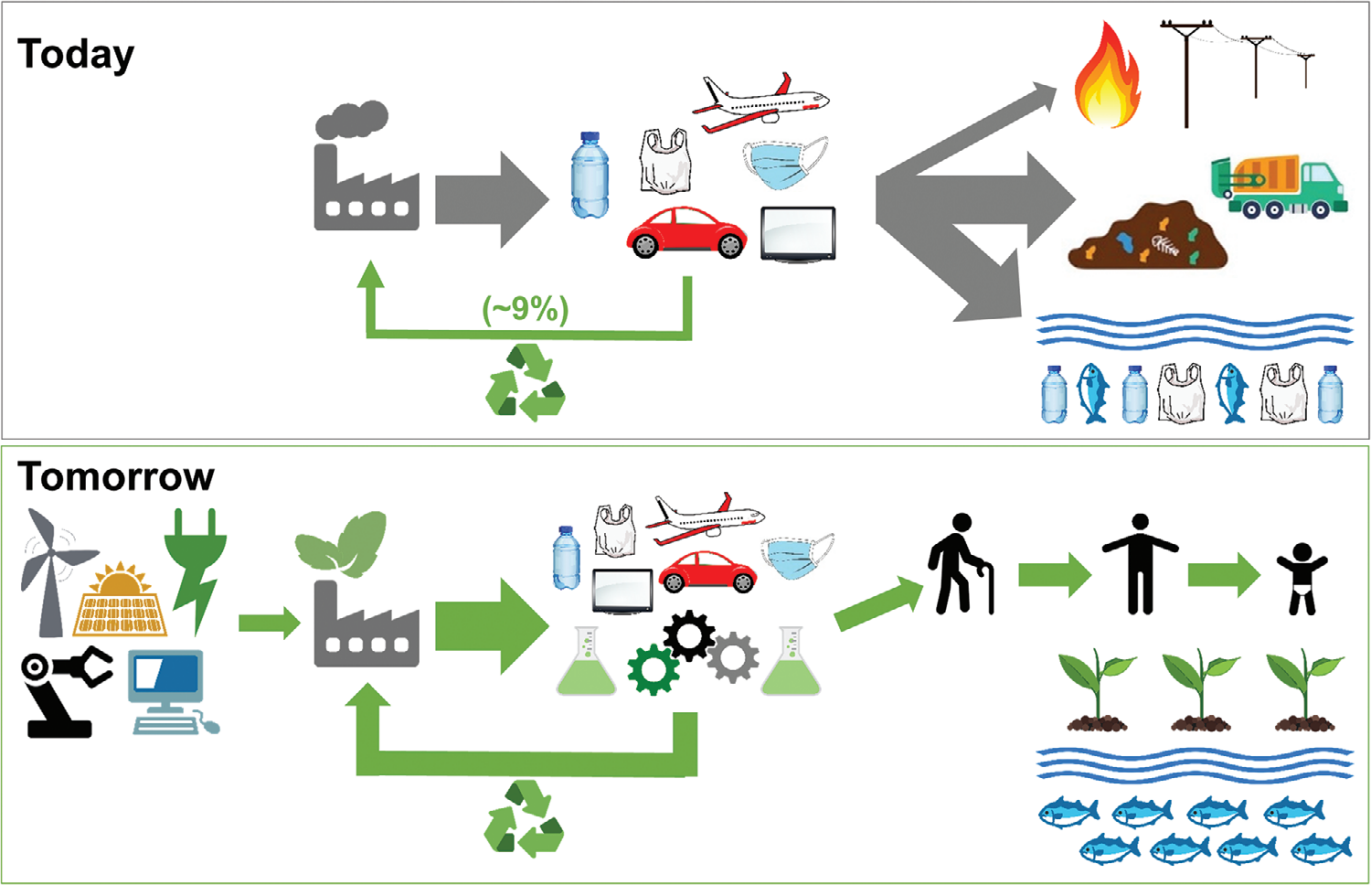
Present and future of polymer industry and its impact on the planet.

The second century of polymer science brings enormous challenges and opportunities for polymer scientists, who must design methods and materials that respond to the evolving needs of our society and planet (**Scheme** **1**). It is of utmost importance to shift the focus from performance to performance and sustainability, when developing or improving polymer syntheses and products.^[^ ^10^ ^]^ Polymer chemists have several tools to address these challenges, and among them are certainly the principles of green chemistry and the ability to precisely control polymerization via reversible deactivation radical polymerization (RDRP) techniques.

## 2 Reversible Deactivation Radical Polymerization

Molecular weight, dispersity, as well as molecular architecture (composition, topology, and functionality) are the most important factors that influence the properties and applications of polymers. Growing demand for functional polymeric materials having specific properties advanced the development of innovative and effective modification methods. In this context, macromolecular engineering enables the design of polymers for specific applications via precision synthesis and thorough characterization.^[^ ^11^ ^]^

Controlled/living polymerization is one of the most important methods for the synthesis of polymers with well‐defined and predictable properties.^[^ ^12^ ^]^ This method includes all chain‐growth polymerizations such as cationic, anionic, ring‐opening, transition metal‐catalyzed, and radical polymerizations. The term “living polymer” was coined for the first time in 1956 by Szwarc.^[^ ^13^ ^]^ To be considered “controlled/living,” a polymerization process must be characterized by fast and efficient initiation, concurrent growth of all chains, and negligible contribution from both irreversible chain termination and chain transfer.^[^ ^14^ ^]^

**Figure 1:**
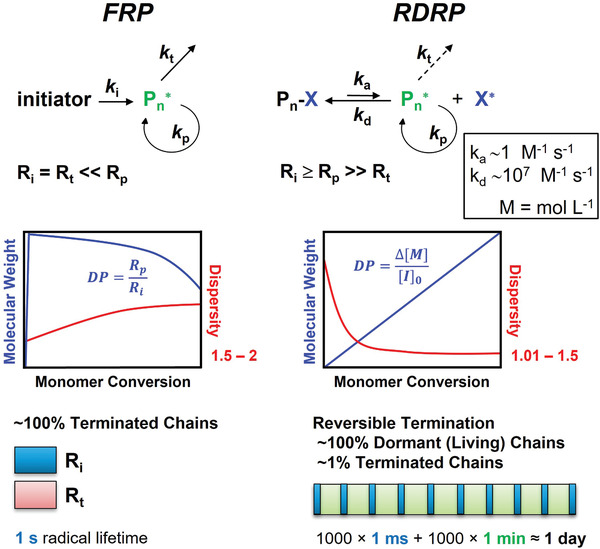
Comparison between conventional free radical polymerization (FRP) and reversible deactivation radical polymerization (RDRP). *R*_i_, *R*_t_, and *R*_p_ are the rate of initiation, termination, and propagation; *k*_i_, *k*_t,_ and *k*_p_ are the corresponding rate constants; *k*_a_ and *k*_d_ are the radical activation and deactivation rate constants. DP is the degree of polymerization, M is the monomer, and I the initiator.

Radical polymerizations benefit from a broad monomer scope and tolerance to many solvents, including water, as well as many functional groups. However, a true living radical polymerization is prevented by inevitable bimolecular radical terminations. The development of controlled radical polymerization (CRP) based on establishing a dynamic equilibrium between propagating radicals and dormant species that cannot propagate and terminate, allowed for extending the lifetime of growing polymer chains from *ca*. 1 s to several hours or days (**Figure** **1**). Consequently, the composition, topology, and functionality of synthesized materials can be precisely controlled. In CRPs, dormant species can be reactivated to propagating radicals that are deactivated to their dormant state after adding a few monomer units, and therefore the process resembles a living polymerization.^[^ ^12a^ ^]^ Alternatively, radicals can rapidly exchange with efficient transfer agents via degenerative transfer.^[^ ^15^ ^]^ Following IUPAC recommendation, these processes are collectively termed reversible deactivation radical polymerizations (RDRPs). Figure 1 shows a comparison between conventional free radical polymerization (FRP) and RDRP methods.

**Figure 2:**
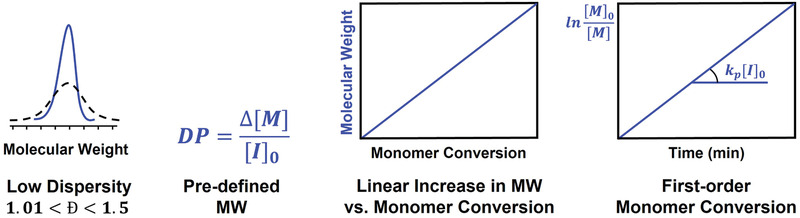
Requirements of controlled radical polymerization.

Well‐controlled polymerization processes should meet the following requirements (**Figure** **2**): i) the obtained polymer has low dispersity (*Đ*, although there are processes in which intentional high dispersity and broad MWD is targeted) and pre‐defined molecular weight (MW), which can be expressed as the number‐average degree of polymerization (DP), determined by the ratio of the concentration of converted monomer to the initial concentration of initiator (DP  =  Δ[M]/[I]_0_), ii) linear increase in polymer MW with conversion, and iii) linear first‐order kinetic plot.^[^ ^12a^ ^]^

The last two decades witnessed a revolution in polymer synthetic chemistry, with the development of several RDRP techniques,^[^ ^15^, ^16^ ^]^ which in addition were included in the list of 10 emerging technologies of the past century that can change the world.^[^ ^17^ ^]^

## 3 ATRP: General Mechanism and Recent Advances

**Figure 3:**
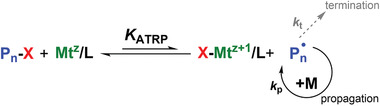
General mechanism of atom transfer radical polymerization (ATRP), where *K*_ATRP_ is the ATRP equilibrium constant.

Atom transfer radical polymerization (ATRP) is one of the most powerful and versatile RDRP techniques, developed in 1995 by Wang and Matyjaszewski^[^ ^16a^ ^]^ and Sawamoto.^[^ ^16c^ ^]^ In ATRP the equilibrium between active radicals and dormant species is achieved through a concurrent atom and electron transfer reaction regulated by a catalyst, which is typically a transition metal complex (**Figure** **3**).^[^ ^18^ ^]^ The low‐oxidation‐state metal complex, Mt^z^/L (Mt^z^ is the metal ion in oxidation state z, and L is a ligand), acting as an activator, reversibly reacts with an alkyl halide (RX) initiator or halogen (X)‐capped dormant chain. This reaction generates radicals and the corresponding high‐oxidation‐state metal complex with a coordinated halide ligand, XMt^z+^ ^1^/L, which then acts as deactivator, reverting the radical back to its dormant state. Therefore, ATRP proceeds through a series of activation and deactivation cycles, ending upon complete monomer consumption or deliberate deactivation of the system. The ATRP equilibrium is strongly shifted towards the dormant species, thus the concentration of growing macroradicals is low and termination reactions are minimized (<5% of polymer chains terminate).^[^ ^18a^ ^]^

The ATRP equilibrium can be established by starting either from the low‐oxidation‐state metal complex and RX (normal ATRP), or from the high‐oxidation‐state metal halide complex and a radical source (reverse ATRP). The latter facilitates the reaction handling since the starting metal complex is air stable.^[^ ^19^ ^]^ Simultaneous reverse and normal initiation (SR&NI) ATRP can be achieved by employing a conventional radical initiator, RX, and a high‐oxidation‐state metal halide complex. This method enables use of more active catalysts, thus decreasing the catalyst loading from >10 000 to <1000 parts per million (ppm, relative to the molar concentration of monomer).^[^ ^20^ ^]^

**Figure 4:**
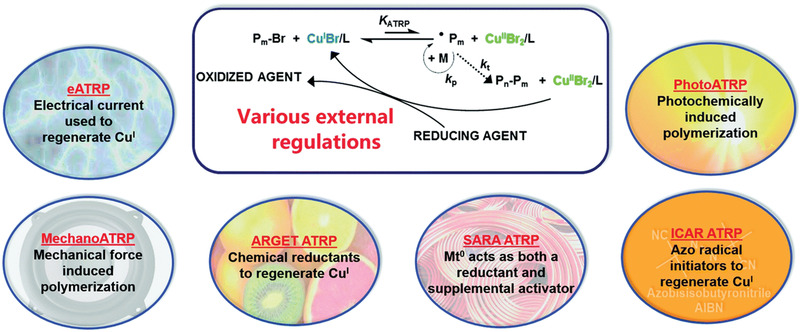
ATRP techniques with continuous regeneration of Cu^I^/L activator. Reproduced with permission.^[^ ^27^ ^]^ Copyright 2018, Royal Society of Chemistry.

Aiming to develop more efficient, environmentally friendly and scalable techniques, several methods were discovered in which a low catalyst loading could be used (<100 ppm). These ATRP methods (**Figure** **4**) proceed via continuous regeneration of the activator form of the catalyst using a chemical reductant, e.g., ascorbic acid (AGET and ARGET, activators (re)generated by electron transfer ATRP),^[^ ^21^ ^]^ or a radical initiator, e.g., AIBN, azobisisobutyronitrile (ICAR, initiators for continuous activator regeneration),^[^ ^22^ ^]^ metallic copper (SARA, supplemental activator and reducing agent ATRP),^[^ ^23^ ^]^ photoinitiation (*photo*ATRP, photochemically mediated ATRP)^[^ ^24^ ^]^ electric current or potential (*e*ATRP, electrochemically mediated ATRP),^[^ ^25^ ^]^ or ultrasound (*mechano* and *sono*ATRP).^[^ ^26^ ^]^

The efficiency of ATRP primarily depends on the appropriate selection of RX initiator and catalyst. The concentration of RX determines the number of growing chains, provided that the initiation efficiency is high. Typical ATRP initiators contain groups that stabilize the generated radicals (e.g., benzyl, ester, nitrile groups), and their reactivity is inversely proportional to the bond dissociation energy (BDE) of the C—X bond, which depends on the nature of the stabilizing group and the halogen atom.^[^ ^16^, ^18^, ^28^ ^]^ The initiation efficiency depends on the rate and selectivity of migration of the halogen between the growing chain and the catalytic complex. The most often used initiators are benzyl halides, *α*‐haloesters, haloalkanes, haloketones, haloamides, and halonitriles, where X = Br or Cl. Alkyl iodides have lower BDE than corresponding RBr or RCl, but the affinity of iodides for the transition metal complex is generally too low. Alkyl fluorides are much less reactive due to the much greater BDE of C—F bonds, as compared to C—Br or C—Cl. Depending on the number of initiating sites (C—X moieties) in the (macro)initiator, polymers with telechelic, star or grafted structure can be obtained.

The ATRP catalytic complex regulates the equilibrium between active and dormant species. The oxidized transition metal in the catalytic complex should have high affinity for the halide ion (i.e., halidophilicity) and should possess two stable oxidation states separated by one electron. Transition metals used in ATRP include titanium, rhenium, iron, ruthenium, osmium, rhodium, cobalt, nickel, palladium, and copper, the latter being the most common.^[^ ^29^ ^]^ Typical ligands are polydentate alkylamines and pyridine derivatives. In normal ATRP, the most popular catalysts are Cu complexes with ligands such as 2,2’‐bipyridine (bpy), 4,4′‐di(5‐nonyl)‐2,2′‐bipyridine (dNbpy), N,N,N′,N”,N”‐pentamethyldiethylenetriamine (PMDETA) or 1,1,4,7,10,10‐hexamethyltriethylenetetramine (HMTETA). In ATRP with low ppm catalyst loading, the most common ligands for Cu catalysts are tris[2‐(N,N‐dimethylamino)ethyl] amine (Me_6_TREN) and tris(2‐pyridylmethyl)amine (TPMA). The selection of the ligand is crucial because it determines the activity of the catalytic complex and affects its solubility in the reaction environment.^[^ ^16^, ^30^ ^]^ The activity of the catalytic system depends on the number and type of nitrogen atoms, number of carbon atoms between nitrogen atoms,^[^ ^31^ ^]^ and both steric and electronic effects.^[^ ^32^ ^]^ Besides the catalyst and initiator, the efficiency of ATRP depends on many other parameters affecting the reaction equilibrium, such as the type of solvent, reaction temperature, and the ratio between the system components.

**Figure 5:**
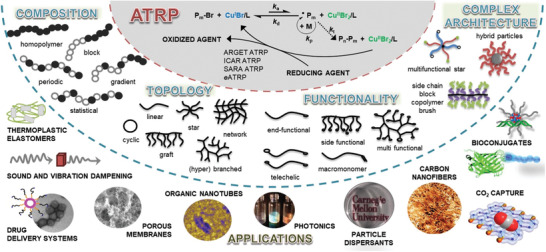
Overview of ATRP advancements with ppm amounts of copper catalysts, engineering of various macromolecular architecture and applications of resulting materials. Reproduced with permission.^[^ ^11b^ ^]^ Copyright 2014, American Chemical Society.

The classical ATRP method has some limitations including the use of a high concentration of an air‐sensitive catalyst, and the consequent necessity to remove the metal species from the final products, particularly for biomedical applications. The development of low‐ppm ATRP techniques allowed for diminishing the amount of catalyst, increasing the oxygen tolerance of the polymerization, and developing systems to conduct ATRP in open air.^[^ ^33^ ^]^ These advances led to the preparation of a large variety of (co)polymers with precisely controlled architectures, therefore relevant to a broad range of applications, from commodities to advanced and specialty materials. Ultimately, the (co)polymers obtained by ATRP are used as components of coatings, elastomers, surfactants, lubricants, dispersants, and for applications in medicine and electronics (**Figure** **5**).^[^ ^11b^ ^]^

ATRP has been successfully employed in industry, particularly upon the creation of a CRP Consortium at Carnegie Mellon University, which has comprised 60 member companies, and issued 65 US patents and 17 commercial licenses.^[^ ^34^ ^]^ The market value of polymeric materials obtained by ATRP and other RDRP methods was predicted to reach about 20 billion USD a year.^[^ ^35^ ^]^ The success of ATRP and other RDRP techniques and their use for commercial products lead to the need for minimizing the environmental impact of the synthetic protocols. Moreover, these techniques offer unprecedented opportunities for tailoring polymer architecture and functionalities, which must be exploited to make innovative green materials.

## 4 The 12 Principles of Green Chemistry and Their Implementation in ATRP

To date, the broad applications of ATRP and RDRP, in general, are limited by some potential environmental concerns. These include the toxicity of monomers, catalysts, and additives, the high amount of energy needed for production processes, as well as the transfer from a lab‐scale to macro‐scale production. Therefore, recent advances in RDRPs have greatly focused on increasing the sustainability of these processes and their products.

In the pursuit of more sustainable polymerizations and polymers, it is mandatory to implement approaches consistent with the principles of green chemistry, aligned with responsible design and development of products and processes. According to the US Environmental Protection Agency (EPA), green chemistry refers to “innovative chemical technologies that reduce or eliminate the use and generation of hazardous substances.”^[^ ^36^ ^]^ In 1998, Anastas and Warner delineated 12 principles that should be adopted by all chemists as guidelines to improve existing procedures, create more sustainable ones and produce innovative materials.^[^ ^37^ ^]^ The principles of green chemistry focus on the environmental impact of chemicals in their intended use (product design) and manufacturing footprint (process design).

The 12 principles of green chemistry are summarized as follows:

P1: Prevent waste.
P2: Design methods to maximize the incorporation of all substrates used in the chemical process into the final product (atom economy).
P3: Design less hazardous chemical synthesis.
P4: Design safer chemicals and other products.
P5: Use safer solvents and auxiliaries.
P6: Design for energy efficiency.
P7: Use renewable feedstocks.
P8: Avoid derivatization because it requires additional reagents and can generate waste.
P9: Use selective catalysts, not stoichiometric reagents.
P10: Design degradable products.
P11: Analyze process in real‐time to prevent pollution.
P12: Minimize the potential for accidents.

Green methods in chemistry refer mainly to the use of environmentally friendly (nontoxic) and reusable reagents and solvents, as well as effective catalysts, and to the development of technologies that consume less energy. Alongside, green chemistry refers to the generation of products that achieve desired performance while having a little environmental impact, thus being recyclable, or degradable, or derived from abundant and benign sources.

The present review provides a broad overview of the implementation of the 12 principles of green chemistry in ATRP. It is noteworthy that the ATRP technology received the Presidential Green Chemistry Challenge Award in 2009. While some green aspects of ATRP were described in the previous reviews,^[^ ^38^ ^]^ during the last 10–15 years ATRP has become much more sustainable and increasing efforts were directed to address environmental challenges. The most recent developments of ATRP that diminished its environmental impact and enhanced its green character are reviewed herein. In particular, the aspects of ATRP that adhere to each principle of green chemistry are described with relevant examples. Several features of ATRP address more than one principle at the same time. However, in the following sections, each topic is assigned to the most pertinent principle to simplify the discussion.

The ability of the most important and environmentally‐friendly features of ATRP to fulfill different principles is highlighted in **Table** **1**, which summarizes the state‐of‐the‐art of green ATRP and indicates the principles where more research efforts are needed.

**Table 1:** Green features of ATRP and their correspondence to the 12 principles of green chemistry (Numbers indicate the level of development of a certain feature and relevance for a specific principle, in ascending order from 0 (not relevant/underdeveloped) to 3 (highly relevant/well‐developed)

Principles	Green features of ATRP													
	Catalytic system				Conditions				Monomers		Materials			
	Ppm loading, high activity	Activator regeneration	Temporal/spatial control	Catalyst recycling	Aqueous media	Oxygen tolerance	High monomer conversion	Bulk	Scope	Biobased	Segmented copolymers, thermoplastic elastomers	Surface modification, hybrids, dispersants	Bioconjugates	Degradable polymers
P1. Prevent waste	3	3	1	2	3	1	3	3	1	1	2	1	1	2
P2. Atom economy	2	2	1	2	2	0	3	3	2	0	1	1	1	0
P3. Less hazardous synthesis	2	3	3	1	3	2	0	1	1	1	0	0	1	0
P4. Benign chemicals	1	1	0	1	2	1	0	0	1	2	3	3	3	3
P5. Benign solvents & auxiliaries	1	3	0	0	3	1	0	0	2	2	1	1	2	0
P6. Energy efficiency	1	3	2	2	3	2	1	0	0	0	1	1	1	2
P7. Renewable feedstocks	0	0	0	0	0	0	0	0	1	3	2	2	2	2
P8. Reduce derivatives	0	0	0	2	0	0	0	0	3	0	1	2	1	2
P9. Catalysis	3	3	1	3	2	2	1	2	0	0	2	2	2	2
P10. Design for degradation	0	0	1	0	0	0	0	0	3	2	2	1	1	3
P11. Real time analysis	0	1	2	0	1	1	1	0	1	0	0	0	0	0
P12. Accident prevention	2	3	3	1	2	2	0	0	0	1	0	0	0	0

### 4.1 P1: Prevent Waste

Chemical processes should use minimal amounts of reagents to reduce the production of waste. The waste generation in ATRP can be decreased by i) maximizing monomer conversion, ii) eliminating solvents, in particular by replacing them with bulk polymerizations, and iii) minimizing the catalyst concentration. In addition, ATRP can be run to limited monomer conversion with recovery and reuse of the unreacted monomer, which can occur by conducting the polymerizations in continuous flow reactors (cf. P6: Design for energy efficiency).^[^ ^39^ ^]^ Therefore, preventing waste enables to eliminate costly and energy‐intensive separation and purification procedures, improving the overall energy efficiency.

#### 4.1.1 High Monomer Conversion in Bulk Polymerizations

**Figure 6:**
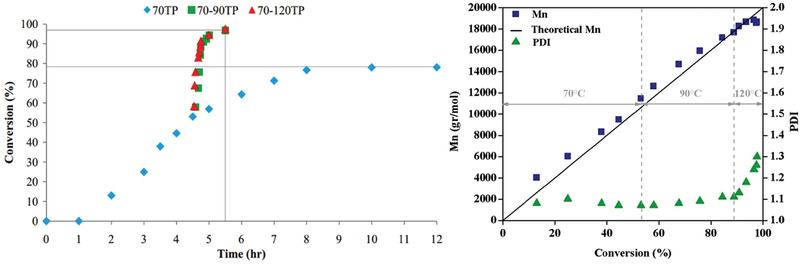
Kinetics of bulk ICAR ATRP of MMA. [MMA]_0_:[EBPA]_0_:[CuBr_2_]_0_:[dNbpy]_0_:[AIBN]_0_:[TBP]_0_ = 200:1:0.05:0.1:0.2:0.2. Reproduced with permission.^[^ ^44^ ^]^ Copyright 2014, Wiley.

The first reported ATRP targeted the bulk polymerization of styrene.^[^ ^12^, ^16^ ^]^ During controlled bulk radical polymerization all chains start growing at the same time and the chain length increases linearly with conversion. If high monomer conversion is reached, the amount of waste is further minimized. However, during the reaction progress, the viscosity increases due to increasing polymer molecular weight and concentration, as well as decreasing amount of monomer, eventually hampering the ability of the system to reach complete monomer conversion. In conventional radical polymerizations, when high monomer conversion is approached, uncontrolled acceleration of the polymerization may occur due to a significant reduction in radical termination rate in highly viscous systems (gel effect or Trommsdorff‐Norrish effect).^[^ ^40^ ^]^ Since in FRP a steady‐state concentration of radicals is established by the balance between rates of initiation and termination, a drop in the latter results in increased concentration of radicals that accelerate propagation, which in turn can lead to an uncontrolled (explosive) process when the heat transfer becomes inefficient. Moreover, an increase in temperature leads to faster thermal radical decomposition, which produces more radicals and further accelerates polymerization. Therefore, proper control of polymerization conditions must be ensured, considering that the efficiency of heat transfer depends on the reaction volume, viscosity, size and shape of the reactor, and efficiency of mixing (cf. P12: Accident prevention).^[^ ^41^ ^]^ ATRP seldom exhibits a gel effect.^[^ ^42^ ^]^ Although at high conversion the increased viscosity leads to decreased termination rate and increased *R*_p_/*R*_t_ ratio, the concentration of radicals in ATRP is established by activation‐deactivation equilibrium and not by a steady‐state between initiation and termination. High conversion of methyl methacrylate (MMA) in bulk ICAR ATRP was achieved with a binary system of two radical initiators, i.e., AIBN, and *tert*‐butyl peroxybenzoate (TBPB) or *tert*‐butyl peroxide (TBP), which decomposes at higher temperature than AIBN.^[^ ^22^, ^43^ ^]^ As such, PMMA was synthesized using ethyl (*α*‐bromophenyl)acetate (EBPA) as initiator and Cu/dNbpy as catalyst, reaching 98% monomer conversion in less than 5 h, with *Đ* ≈1.3 and retained living character via utilization of a step temperature profile (70–90–120 °C, **Figure** **6**).^[^ ^44^ ^]^

#### 4.1.2 High Monomer Conversion in Solution Polymerizations

Nearly quantitative monomer conversion in ATRP can be limited by mass transport phenomena. In fact, the high viscosity of ATRP systems at high conversion can slow down the activation and deactivation kinetics. This can result in increased polymer dispersity at high conversion, despite termination reactions are also suppressed. Performing ATRP under high pressure can help overcoming these limitations. Nearly quantitative conversion in the ATRP of styrene under high pressure was obtained, forming polystyrene with ultrahigh molecular weight (UHMW).^[^ ^45^ ^]^ Indeed, the design of UHMW polymers requires polymerization conditions where *k*_p_ is maximized while *k*_t_ and *k*_tr_ (chain transfer rate coefficient) are both suppressed. It is also challenging to prepare star polymers with high MW, due to star‐star coupling reactions. Recently, high molecular weight (*M*_w_ > 300 000) star‐branched polymers with controlled (*Đ* < 1.08) architecture were efficiently (conversion > 95%) obtained via SARA ATRP.^[^ ^46^ ^]^ By controlling the size and number of initiating sites and sterics of acrylic monomers, the star‐star coupling was minimized.

### 4.2 P2: Atom Economy

The concept of atom economy (or atom efficiency) refers to “chemical reactions that do not waste atoms”.^[^ ^47^ ^]^ Ideally, all materials introduced in the system should be converted into the final products. Such high synthetic efficiency would require the reactions not only to be devoid of any side products, but also to exhibit high chemo‐, regio‐, stereo‐, and enantioselectivity. In general, a shift of focus from the mere reaction yield, to avoiding any waste in the form of other reactants (e.g., solvent, catalyst, additives) not incorporated in the final product must be emphasized. While atom economy initially focused on small molecules, several concepts have been transferred to the area of polymeric materials significantly affecting the economic consequences. Nowadays atom economy is widely expressed by using the environmental factor (E‐factor) denoted as (kg waste)/(kg product).^[^ ^48^ ^]^

Substrates (initiator and monomers) in ATRP are fully incorporated in the final product, assuming quantitative monomer conversion (cf. P1:Prevent waste). In addition, the concentration of the catalyst must be low to further enhance the atom economy. Low‐ppm catalyst loading became possible with the introduction of AGET, ICAR, and ARGET methods, which resulted in the reduction of copper concentration down to hundreds or tens of ppm.^[^ ^21^ ^]^ The extent of side reactions and thus the selectivity of an ATRP process strongly depends on the selection of the catalyst. On the other hand, the control on polymer tacticity in ATRP is challenging and generally requires suitable additives.

#### 4.2.1 Mitigation of Side Reactions

Unavoidable termination reactions, e.g., radical–radical coupling, and side reactions^[^ ^16b^ ^]^ that decrease the atom economy can be minimized by tuning the polymerization conditions, including the nature of catalyst, solvent, and (macro)monomer. For example, the formation of R‐Cu^II^/L organometallic intermediates in ATRP can lead to the Cu‐catalyzed radical termination (CRT) process. These intermediates can activate/deactivate radicals as in an organometallic mediated radical polymerization (OMRP), and they can further react with radicals to regenerate the Cu^I^/L activator, but at the expense of a radical termination event. CRT can be the dominant termination mode in ATRP of methyl acrylate (MA), being *ca*. 40 times faster than bimolecular radical–radical terminations, accounting for up to 95% of terminated chains.^[^ ^49^ ^]^ This phenomenon can be mitigated to a certain extent by using more active catalysts, resulting in lower concentration of Cu^I^ activator and thus limited generation of R‐Cu^II^/L intermediate.^[^ ^50^ ^]^ Furthermore, the formation of organometallic species is more detrimental in Fe‐catalyzed ATRP.^[^ ^51^ ^]^ However, more efforts are needed to understand these side reactions and guide the design of more selective ATRP catalysts.

**Figure 7:**
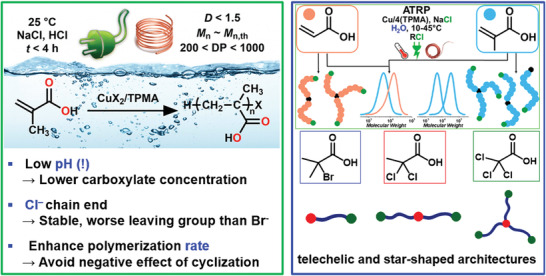
ATRP of methacrylic acid in water under electrochemical and SARA conditions (green frame); extension of the procedure to acrylic acid and different macromolecular architectures (blue frame). Reproduced with permission.^[^ ^52^ ^]^ Copyright 2016, American Chemical Society.

Another side reaction that could be effectively minimized was an intramolecular lactonization reaction of propagating chains during the ATRP of (meth)acrylic acid in water.^[^ ^52^ ^]^ The undesired cyclization resulted in loss of chain‐end functionality and low conversion. These problems were solved by employing strongly acidic pH, alkyl chloride initiators, and by increasing the polymerization rate. Therefore, *e*ATRP and SARA ATRP under optimized conditions gave well‐defined poly(acrylic acid) and poly(methacrylic acid) (PAA and PMAA, respectively) with linear, telechelic, and star‐shaped macromolecular architectures (**Figure** **7**).

#### 4.2.2 Tacticity

In ATRP, controlling the polymer tacticity is challenging compared to anionic or coordinative polymerizations, since the formed prochiral radical center diffuses away from the catalyst before the next monomer unit can be added. Somewhat efficient approaches focus on the addition of Lewis acid and/or chiral ligands, which however decrease the atom efficiency when used in high amounts. Well‐defined homopolymers of *N*,*N*‐dimethylacrylamide (DMAA)^[^ ^53^ ^]^ and *N*‐hydroxyethyl acrylamide (HEAA)^[^ ^54^ ^]^ with 80–85% proportion of *meso* dyads were prepared by ATRP in the presence of yttrium(III) trifluoromethanesulfonate, Y(OTf)_3_. In addition, one‐pot synthesis of atactic‐*b*‐isotactic PDMAA‐*b*‐PHEAA stereoblock copolymers was achieved for the first time by adding Y(OTf)_3_ at a specific conversion. Syndiotacticity‐enhanced ATRP of MMA, with the tacticity resulting from the chiral side‐armed bisoxazoline (SaBOX) ligand/copper catalytic system was reported.^[^ ^55^ ^]^ Moreover, polymer tacticity can be controlled by performing ATRP in chiral ionic liquids (cf. P5: Benign solvents and auxiliaries).

### 4.3 P3: Less Hazardous Synthesis

The third green chemistry principle focuses on mitigation of the environmental and health impact that is associated with the chemical process and its products. In polymerization processes, hazards are inherently associated with the exothermic character of polymerizations, as well as with toxicity of the molecular building blocks and reagents.^[^ ^56^ ^]^ Moreover, residual metal catalyst eventually present in the polymer is hazardous for certain applications. The potential threat of exothermicity and ways to prevent associated accidents are discussed in P12: Accident prevention.

#### 4.3.1 Toxicity

To minimize the toxicity of a polymerization process, all the needed compounds should have low toxicity and they should be incorporated into the polymer product or easily removed (to an acceptable concentration). Any potential toxicity of reagents involved in ATRP should be accurately evaluated.^[^ ^57^ ^]^ Polymers exhibit significantly reduced adverse feature relative to monomers. However, the common usage of plastics and long‐term exposure as a result of their ubiquity create considerable threats. Mammals’ kidney filtration is limited by the molar mass and shape of a water‐soluble polymer, with the rough cutoff at *M*_n_ ≈ 30 000, resulting in much longer circulation time in blood above that threshold.^[^ ^58^ ^]^ Pharmaceuticals based on PEGylated species (PEG: poly(ethylene glycol)) are very common and approved by the US Food and Drug Administration (FDA). However, prolonged contact with PEGylated species can lead to the formation of anti‐PEG antibodies, with reported negative health outcomes.^[^ ^59^ ^]^ Therefore, materials that act as PEG alternatives were developed,^[^ ^60^ ^]^ such as zwitterionic polymers, polymethacrylates with short PEG side chains (PEGMA), polyaminoacids, polyacrylamides, polycarbonates, poly(glycerols), or poly(*N*‐vinylpyrrolidone). ATRP was used to grow PEGMA from chymotrypsin‐appended initiators,^[^ ^61^ ^]^ and *ε*‐caprolactone/PEGMA hyperbranched stars exhibiting significantly reduced adverse effects.^[^ ^62^ ^]^ The water‐soluble polymeric analog of DMSO, poly(2‐(methylsulfinyl)ethyl acrylate) (polyMSEA), prepared by ATRP is also envisaged as a promising PEG alternative.^[^ ^63^ ^]^

Eventual toxicity in ATRP products can arise from the contamination of residual catalyst or other reaction components. It should be noted that common and effective reducing agents used in ARGET ATRP are either FDA approved (e.g., tin(II) 2‐ethylhexanoate) or environmentally benign and nontoxic (e.g., glucose or ascorbic acid).^[^ ^64^ ^]^ Moreover, the use of external stimuli, particularly electricity^[^ ^25^ ^]^ and ultrasounds^[^ ^26^ ^]^ allows for avoiding the addition of chemicals into the system to promote the regeneration of the activator, thus eliminating byproducts. To minimize the contamination of the catalyst in the polymer product it is helpful to perform ATRP using very low loading of the Cu catalyst. TPMA scaffolds with strongly electron‐donating groups (e.g., NMe_2_, pyrrolidine, piperidine, morpholine) in *para* position on the pyridyl rings were synthesized to obtain Cu catalysts with very high ATRP activity, up to 9 orders of magnitude more active than the first employed ATRP catalysts.^[^ ^65^ ^]^ These highly active catalysts enabled to perform well‐controlled ICAR ATRP of acrylates with only 10 ppm of copper complexes, therefore substantially diminishing the impact of the catalyst.^[^ ^66^ ^]^ Reduced contamination of Cu in the final polymer can also be achieved with the addition of an isocyanide quenching agent^[^ ^67^ ^]^ (cf. P12: Accident prevention). Moreover, ATRP catalysts based on Fe complexes have enhanced bio‐compatibility, and organocatalysts used in metal‐free ATRP likely present lower toxicity (cf. P9: Catalysis).

#### 4.3.2 Oxygen‐Tolerant Processes

**Figure 8:**
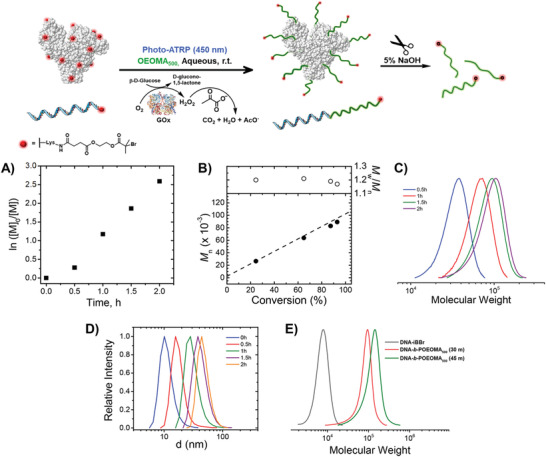
“Breathing” ATRP with enzyme‐induced deoxygenation. Grafting from ATRP initiator‐modified protein (BSA) and DNA with *photo*ATRP under 450 nm irradiation. (A) Semilogarithmic kinetic plot, (B) evolution of *M* *_n_* and *M* *_w_* */M* *_n_* with conversion, (C) Gel permeation chromatography (GPC) traces of cleaved polymers, and (D) evolution of protein–polymer conjugate particle size over the reaction progress, measured by dynamic light scattering. (E) GPC traces of DNA macroinitiator (DNA‐iBBr) and DNA‐pOEOMA_500_ conjugates after 30 and 45 min of reaction. Reproduced with permission.^[^ ^69^ ^]^ Copyright 2018, American Chemical Society.

The simplification of synthetic procedures and equipment can decrease associated hazards, and thus oxygen‐tolerant polymerizations are inherently safer processes. The requirement for deoxygenation that is common to all RDRPs to prevent reaction quenching by molecular oxygen results in the use of an inert gas source and limits the application of the technique.^[^ ^68^ ^]^ In ATRP, partial oxygen tolerance can be achieved by using various reducing agents (e.g., Cu^0^, Fe^0^, ascorbic acid) or photoirradiation, however, these strategies do not allow for operating in open‐to‐air conditions.^[^ ^18^, ^27^ ^]^ The utilization of glucose oxidase (GOx) and sodium pyruvate (SP) led to the so‐called “breathing ATRP”^[^ ^33^, ^69^ ^]^ that could be performed in open air (**Figure** **8**).

Inspired by this work, other fully oxygen tolerant and environmentally‐benign systems were developed: i) the solely SP‐based PICAR ATRP process,^[^ ^33c^ ^]^ which can be conducted in both water and organic solvents, while the GOx/SP system is limited to aqueous media; and the ii) “oxygen‐fueled ATRP”,^[^ ^70^ ^]^ where a nanomolar biocatalytic GOx/horseradish peroxidase (HRP) system drives the controlled polymerization while consuming O_2_. Moreover, a robust ATRP methodology was developed that is applicable to the synthesis of protein–polymer bioconjugates in an oxygen‐tolerant manner under photoinduced conditions.^[^ ^71^ ^]^ Polymerizations were performed in plastic syringes without any additives nor deoxygenation procedures, using light irradiation ranging from UV to blue light and sunlight, and ppm amounts of a copper catalyst. The oxygen tolerance was achieved by harnessing a microbial metabolism process, in which O_2_ was consumed by aerobic respiration followed by electron donation to the Cu^II^ species to trigger the polymerization.^[^ ^72^ ^]^ As such, oxygen‐tolerant polymerizations largely improve the viability and simplicity of the applied methodology, as well as the nonhazardous and reliable character of the chemical syntheses.^[^ ^33d^ ^]^

### 4.4 P4: Design Benign Chemicals

The precise control over polymer architecture and topology achieved in ATRP, and the possibility to grow polymers from a broad variety of functionalized surfaces via surface‐initiated ATRP (SI‐ATRP) enable the preparation of well‐defined, functional polymer and hybrid materials. The synthesis and applications of hybrid materials made by ATRP were described in recent reviews.^[^ ^73^ ^]^ ATRP has been widely employed to prepare materials for biomedical applications, including polymer conjugates with proteins, nucleic acids, carbohydrates, and cells.^[^ ^66^, ^74^ ^]^ These biohybrid materials enhanced the stability and functionality of biomacromolecules, ultimately enabling their use for cellular and tissue engineering. Additionally, ATRP has been used to design biosensors, drug delivery systems, hydrogels for tissue engineering, and structurally tailored and engineered macromolecular (STEM) gels.^[^ ^75^ ^]^ The latter are inimer‐decorated networks amenable to facile postmodification, therefore good precursors for 3D printing of artificial tissues, actuators, or wearable devices.

ATRP is also a powerful technique to design smart materials that possess enhanced durability and longer service life, as well as reusable materials, thus contributing to the reduction of plastic pollution and associated risks for the environment and human health.

#### 4.4.1 Dispersants and Compatibilizers

Copolymers with well‐controlled architectures can act as efficient dispersants and compatibilizers for immiscible substances, thus they can be used to improve compatibility of polymer blends, ultimately eliminating the need for sorting plastic waste.^[^ ^5^, ^7^ ^]^ Compatibilizers are generally blocked or graft copolymers that segregate at the interface of immiscible polymers. Maleic anhydride terminated polystyrene (MA‐PS‐MA) was prepared by ATRP and reacted with the amine end group of nylon 6 in nylon 6/PS melt, forming ternary blends with enhanced mechanical properties compared to the binary nylon 6/PS blend.^[^ ^76^ ^]^ Polyethylene (PE)‐*graft*‐PMMA copolymers were synthesized by metallocene‐catalyzed copolymerization of ethylene and 10‐undecen‐1‐ol, followed by the introduction of the ATRP initiating sites to grow PMMA side chains via grafting from approach.^[^ ^77^ ^]^ Another strategy toward PE‐graft‐PMMA compatibilizers used ring‐opening metathesis polymerization (ROMP) of *cis*‐cyclooctene and cyclooctene functionalized with *α*‐bromoisobutyrate for the PE backbone.^[^ ^78^ ^]^ Strongly improved compatibility of highly immiscible PE/PMMA blends was observed by using only 1 wt% PE‐*graft*‐PMMA with relatively short PMMA chains. While the small loading compensates for the laborious compatibilizer synthesis, more efficient procedures should be defined by using more recent and greener developments of ATRP.

#### 4.4.2 Upcycling of Commercial Polymers

To turn plastic waste into high‐value materials, it is important to be able to modify polymers by introducing functionalities that enable subsequent chemical transformations, or by exploiting existing functionalities or reactive defects. ATRP was employed to graft styrene and various acrylates from poly(vinyl chloride) (PVC) with the chloroacetyl groups acting as initiating sites, by using poly(vinyl chloride)‐*co*‐(vinyl chloroacetate) as a macroinitiator.^[^ ^79^ ^]^ Moreover, ARGET and *photo*ATRP have been used to graft polymers from commercial PVC and poly(vinylidene fluoride) (PVDF) membranes.^[^ ^80^ ^]^ Defects such as tertiary C—Cl functionalities in PVC and unsaturated or allylic C—F sites in PVDF could act as initiating sites. Besides improving the material properties, these modifications could facilitate the sorting and recovery of plastic waste.^[^ ^81^ ^]^ In addition, C—H functionalization strategies hold great promise for commodity polymer upcycling. Recently, a photocatalytic method that operates under mild conditions was implemented to introduce fluorobromoalkyl groups into aromatic polymers, including commercial and post‐consumer polystyrene.^[^ ^82^ ^]^ The inserted groups were used to initiate ATRP and form graft copolymers.

#### 4.4.3 Thermoplastic Elastomers

**Figure 9:**
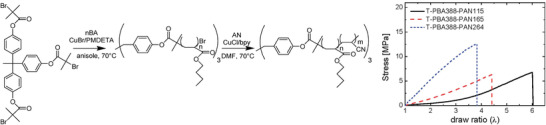
Synthetic procedure to make three‐arm star P*n*BA‐PAN TPEs via ATRP, and effect of PAN fraction on the stress–strain curve. Reproduced with permission.^[^^85b]^ Copyright 2008, American Chemical Society.

Vulcanized rubbers traditionally contain irreversible crosslinks that improve the material properties but prevent their reprocessability. In contrast, thermoplastic elastomers (TPEs) combine the recyclability and processability of thermoplastics with elastomeric character.^[^ ^18b^ ^]^ TPEs can be melt‐processed due to the presence of physical crosslinks as opposed to irreversible chemical crosslinks of conventional rubbers. ATRP was used to prepare TPEs ranging from traditional triblock copolymers built from two hard end‐blocks and a soft central block, to star‐block, grafts, and combs with block side chains. Conventional polystyrene‐based TPEs have low upper service temperature (≈100 °C), which was increased by synthesizing PMMA‐*b*‐PBD‐*b*‐PMMA (BD, butadiene) via tandem ROMP‐ATRP.^[^ ^83^ ^]^ However, this material was susceptible to oxidation due to the presence of unsaturated double bonds. Thus, all acrylic TPEs based on PMMA‐*b*‐P*n*BA‐*b*‐PMMA (*n*BA, *n*‐butyl acrylate) were prepared by ATRP with halogen exchange to ensure efficient re‐initiation of the P*n*BA‐Br macroinitiator.^[^ ^84^ ^]^ Star‐shaped acrylic TPEs demonstrated good tensile strength and tunable properties, while replacing PMMA with polyacrylonitrile (PAN, **Figure** **9**) increased the service temperature, as PMMA block depolymerizes at >250 °C.^[^ ^85^ ^]^ When the PMMA end blocks in linear acrylic TPEs were replaced by a rosin‐derived polymethacrylate, the elongation was preserved, demonstrating that renewable feedstocks can impart comparable properties to fossil feedstocks (cf. P7: Use of renewable feedstocks).^[^ ^86^ ^]^ Lignin and cellulose were also used as renewable platforms to form TPEs by grafting from *P* *n* *BA‐PMMA* blocks.^[^ ^87^ ^]^ Fully biobased TPEs were achieved from various bioderived monomers (e.g., lysine, itaconate, and furfural) by combining ATRP and click chemistry or ROMP.^[^ ^88^ ^]^ Copolymer compositions were optimized to obtain desired, up to 700% elongations.^[^ ^88a^ ^]^

#### 4.4.4 Self‐Healing Polymers

ATRP was used to make self‐healing polymers that can recover from damages extending their service life. Self‐healing ABA triblock copolymers were prepared via ATRP by synthesizing a telechelic PMMA, then extended using an acrylate monomer with an amide in the side chain.^[^ ^89^ ^]^ Hydrogen bonds in the soft segments enabled the TPE materials, upon cut, to completely recover their mechanical properties within 24 h at 60 °C. The same concept was expanded to TPE brushes with a polystyrene backbone and polyacrylate amide side chains.^[^ ^90^ ^]^ In polar solvents the brushes assumed a globular morphology that was retained upon solvent evaporation, forming a material with spontaneous self‐healing capability at room temperature. Recently, Urban et al. demonstrated that simple PMMA/P*n*BA copolymers within a narrow range of composition (50/50 and 45/55 PMMA/P*n*BA) are able to self‐heal, recovering 90–100% tensile strength within 14 h without applying heat or forces.^[^ ^91^ ^]^ The healable nature was attributed to Van der Walls forces originating from interchain interactions promoted by alternating sequences. Numerical simulations of PMMA/P*n*BA copolymers prepared by ATRP under various conditions revealed that the number of alternating sequences is maximized for a statistical copolymer with 1/1 MMA/*n*BA composition.^[^ ^92^ ^]^ Thus, a feeding strategy in which MMA is continuously fed into the polymerization mixture to obtain statistical copolymers should provide materials exhibiting more effective self‐healing ability.

**Figure 10:**
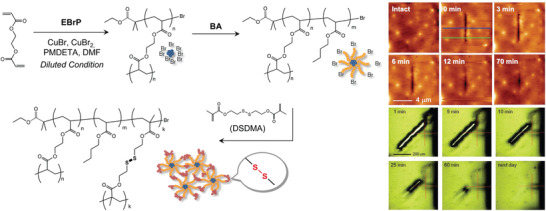
Synthetic route toward SS‐functionalized star polymers via ATRP and demonstration of the self‐healing ability upon cut (upper image series, scale bar 4 mm), and upon penetration (lower image series), as observed by AFM and optical microscopy, respectively. Reproduced with permission.^[^ ^94^ ^]^ Copyright 2012, American Chemical Society.

Dynamic bonds were also introduced in polymer networks made by ATRP to obtain materials with improved mechanical integrity and re‐processability, different from traditional high‐performance thermosets that cannot be remolded. Poly(methacrylic ester)s made by ATRP, containing alkoxyamine units in the side chains underwent cross‐linking upon heating via a radical exchange reaction of the alkoxyamines.^[^ ^93^ ^]^ The resulting gel was reversed to solution upon heating in the presence of excess alkoxyamines. Star polymers containing thiol group at the arm periphery were made by ATRP and cross‐linked to disulfide bonds (SS) by oxidation (**Figure** **10**).^[^ ^94^ ^]^ Under reducing conditions the SS bonds were cleaved and then reformed by oxidation. The ability to self‐heal with no external intervention was demonstrated by atomic force microscopy (AFM). Diels‐Alder (DA) reactions are frequently exploited for dynamic covalent interactions. For instance, biobased furfuryl methacrylate (FMA) served as a reactive diene for the DA reaction in the presence of a bismaleimide dienophile. Thus, a TPE composed of hard PFMA segments and soft poly(2‐ethylhexyl acrylate) could be reversibly cross‐linked by heating and cooling cycles.^[^ ^95^ ^]^ Similarly, reprocessable networks were made by metal‐free ATRP of FMA, cellulose, and fatty acid‐derived monomers, and repeatedly demonstrated recovery of mechanical strength after being cut and remolded for 15 min at 130 °C.^[^ ^96^ ^]^ Polyurethane blocks extended with poly(4‐vinylpyridine) by ATRP and cross‐linked via quaternization with a dibromoalkane exhibited vitrimer‐like properties (stress‐relaxation) and reprocessability.^[^ ^97^ ^]^ SI‐ATRP was employed to grow various polymer brushes from the surface of SiO_2_ nanoparticles, graphene oxide sheets and cellulose nanocrystals via a grafting from approach. The resulting nanocomposite hydrogels exhibited high mechanical strength and autonomous self‐healing, thanks to dynamic supramolecular interactions.^[^ ^98^ ^]^

### 4.5 P5: Benign Solvents and Auxiliaries

Solvents can aid mass and heat transfers during polymerizations and facilitate separations and purifications. However, VOCs (volatile organic compounds) with high flammability and toxicity constitute an environmental concern when used as solvents, therefore non‐toxic and recyclable alternatives should be employed. In ATRP, solvent not only solubilizes reagents and products, but also helps avoid diffusional limitations of ATRP reactions^[^ ^43a^ ^]^ and affects the activity of the catalyst by primarily influencing its activation rate constant (*k*_act_) and the affinity of Cu^II^/L for halides.^[^ ^99^ ^]^ Various green solvents have been used in ATRP to minimize its environmental impact, including water, ionic liquids (ILs), deep eutectic solvents (DESs), and supercritical carbon dioxide (scCO_2_).^[^ ^100^ ^]^ ILs and DESs are greener alternatives to VOCs, mainly due to their negligible vapor pressure and nontoxicity.

#### 4.5.1 Water

Water is likely the greenest solvent and its high heat capacity results in slow cooling or heating of aqueous phases, which prevents sudden thermal runaways in polymerizations (cf. P12: Accident prevention).^[^ ^101^ ^]^ However, the use of water as polymerization solvent is limited by the hydrophobicity of many monomers/polymers, as well as by the moisture‐sensitive nature of many reagents and catalysts. Aqueous ATRP allows for obtaining well‐defined water‐soluble polymers,^[^ ^102^ ^]^ and various protein–polymer bioconjugates.^[^ ^103^ ^]^ However, conducting ATRP in aqueous media faces some challenges. ATRP catalysts exhibit high activity in water, making the process very fast and difficult to control. Indeed, large values of *K*_ATRP_ and *k*_act_ result in a high concentration of radicals and an increased rate of termination reactions. Moreover, the halidophilicity of the X‐Cu^II^/L deactivator is low in aqueous media. Thus, a large fraction of X‐Cu^II^/L is dissociated into halide ions and inactive Cu^II^/L species, leading to decreased concentration of deactivator and inefficient deactivation of propagating radicals, resulting in faster and poorly controlled polymerizations. Moreover, the Cu^I^/L activator can undergo rapid disproportionation.^[^ ^104^ ^]^ Nevertheless, ATRP can be successfully performed in water by employing conditions that shift the equilibrium toward the deactivator, while retaining a sufficient concentration of activator in the reaction medium. These conditions include: i) tuning the Cu^I^ regeneration system to achieve a high molar ratio of Cu^II^ to Cu^I^ species, ii) using an excess of halide salts,^[^ ^23b^ ^]^ iii) using ligands that form complexes with copper with high stability in water (picolylamine‐ and bpy‐based ones),^[^ ^105^ ^]^ and iv) regulating the pH to avoid ligand protonation. The first aqueous ATRP was applied to the synthesis of poly(2‐hydroxyethyl acrylate).^[^ ^106^ ^]^ Since then, ATRP was conducted with a broad variety of water‐soluble monomers,^[^ ^102^ ^]^ such as sodium methacrylate,^[^ ^107^ ^]^ sodium vinylbenzoate (NaVBA),^[^ ^108^ ^]^ 2‐hydroxyethyl methacrylate,^[^ ^109^ ^]^ and oligo(ethylene glycol) monomethyl ether methacrylate (OEOMA).^[^ ^110^ ^]^ However, in systems with a large amount of catalyst (normal, reverse, SR&NI and AGET ATRP) control was generally limited, and polymers of relatively low MW and high dispersity were formed. The development of low‐ppm Cu systems allowed for achieving well‐controlled ATRP in water via SARA ATRP,^[^ ^23^, ^111^ ^]^ *photo*ATRP,^[^ ^112^ ^]^ *mechano*ATRP,^[^ ^26b^ ^]^ *e*ATRP,^[^ ^113^ ^]^ ICAR,^[^ ^22a^ ^]^ and ARGET ATRP.^[^ ^114^ ^]^ The sensitivity of typical ligands for ATRP catalysts to the pH of the medium was exploited to modulate the dispersity of the prepared (co)polymer, ranging from 1.08 to 1.60, while maintaining a high conversion in short reaction time and high chain‐end fidelity.^[^ ^115^ ^]^

#### 4.5.2 Aqueous Dispersions

Aqueous dispersions are often the medium of choice for large‐scale industrial polymerizations.^[^ ^116^ ^]^ Water‐borne ATRP was developed in suspension, dispersion, microemulsion, and miniemulsion.^[^ ^117^ ^]^ Control in ATRP in oil‐in‐water miniemulsion systems was achieved by a proper choice of the catalyst (hydrophobic complex) and surfactant (non‐ionic). Efficient ATRP in miniemulsion requires having both the Cu^I^/L activator and X‐Cu^II^/L deactivator available in the organic droplets, i.e., where the polymerization takes place. Therefore, hydrophobic ligands containing long alkyl chains were designed to effectively reduce the catalyst solubility in the aqueous phase and to bring sufficient Cu species into the organic phase to establish the atom transfer equilibrium. These ligands include dNbpy and bis(2‐pyridylmethyl)octadecylamine (BPMODA) or (4‐methoxy‐3,5‐dimethyl)pyridylmethyl]octadecylamine (BPMODA*), the latter being more active, thus more suitable for low‐ppm systems.^[^ ^118^ ^]^ Recently, a simplified miniemulsion ATRP based on commercial reagents was developed, by combining the hydrophilic catalyst Cu/TPMA and the anionic surfactant sodium dodecyl sulfate (SDS), which resulted in a strong interaction able to locate a large fraction of the catalyst at the interface of SDS‐stabilized monomer droplets.^[^ ^119^ ^]^ Furthermore, the formation of a small fraction of ion pairs between Cu/TPMA and SDS resulted in some catalyst entering the hydrophobic micelles. Therefore, well‐controlled polymerization was achieved through a combination of interfacial and ion‐pair catalysis. This catalytic system was employed to prepare well‐defined polymers using different activator regeneration techniques, including ARGET,^[^ ^120^ ^]^ ICAR,^[^ ^121^ ^]^ *photo*ATRP,^[^ ^122^ ^]^ *e*ATRP,^[^ ^119^ ^]^ *sono*ATRP,^[^ ^123^ ^]^ and even in open‐to‐air systems with enzymatic degassing.^[^ ^121^ ^]^ Importantly the hydrophilic Cu/TPMA was easily removed from final polymers, by diluting the mixtures to promote catalyst migration into the aqueous phase, leaving an extremely low residual Cu concentration (down to 300 ppb) upon simple centrifugation of the diluted system.^[^ ^120^ ^]^ Finally, this simple catalytic system enabled to perform ATRP in a true ab initio emulsion system,^[^ ^124^ ^]^ in which the polymerization starts in the aqueous phase in the presence of a water‐soluble initiator, and after the nucleation step it proceeds in hydrophobic micelles. The partitioning of the catalytic system into different phases was crucial to achieve stable emulsions, even with a solid content of 30–40 wt% and SDS loading below 3 wt%. The scalability of this process should be favored by its environmentally friendly character, and the predominance of emulsion FRP in industry.

#### 4.5.3 Ionic Liquids

ATRP in ILs generally proceeds rapidly and provides polymers with high molecular weight in good yield, while offering easy separation of the product and recovery of the catalyst.^[^ ^125^ ^]^ ATRP was performed in, e.g., 1‐butyl‐3‐methylimidazolium [BMIm][PF_6_]^[^ ^126^ ^]^ or more generally in 1‐alkyl‐3‐methylimidazolium [RMIm][PF_6_]^[^ ^127^ ^]^ The solubility of monomers in ILs depends on the cationic and anionic substituents of the IL, and limited solubility of monomers can help to minimize side reactions and avoid contamination of polymer with monomer and catalyst residues. Moreover, ILs with particular cations and anions (e.g., BMIm cations with dibutyl phosphate anions) can serve both as a solvent and ligand, due to the chelation of anions to transition‐metal catalysts, eliminating the use of external ligands.^[^ ^128^ ^]^ ILs were also used as solvents in reverse ATRP,^[^ ^129^ ^]^ ICAR ATRP,^[^ ^130^ ^]^ ARGET ATRP,^[^ ^131^ ^]^ and AGET ATRP.^[^ ^132^ ^]^ It was observed that the rate of polymerization increases with increasing the length of the alkyl chain in IL cations.^[^ ^133^ ^]^ Due to the poor miscibility of ILs and inorganic bases or organic solvents, further development considered the application of basic ILs in polymerization processes, to replace inorganic bases such as NaOH, Fe(OH)_3_ and Al_2_O_3_ in AGET ATRP.^[^ ^131^ ^]^ Achiral IL‐mediated ATRP (using, e.g., [BMIm][PF_6_]) produced atactic polyacrylates, whereas ATRP with chiral ILs provided well‐defined polymers with a slightly altered tacticity.^[^ ^134^ ^]^ Generally, the replacement of conventional solvents with ILs in radical polymerization allows for reaching higher monomer conversions, and obtaining polymers with higher degree of polymerization, under similar conditions.

#### 4.5.4 Deep Eutectic Solvents

**Figure 11:**
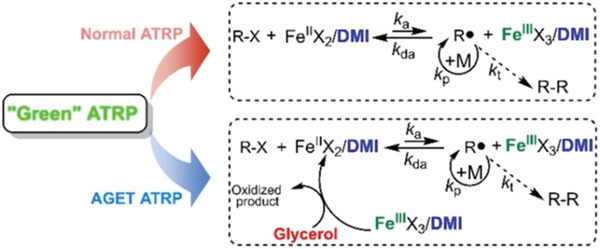
Mechanism of iron‐catalyzed ATRP using 1,3‐dimethyl‐2‐imidazolidinone (DMI) as ligand. Reproduced with permission.^[^ ^140^ ^]^ Copyright 2018, Wiley.

The environmentally friendly properties of ILs have been questioned by the scientific community, thus DESs appear to be a safer alternative, having similar physicochemical properties but exhibiting lower toxicity and cost than ILs.^[^ ^135^ ^]^ Typical components of a DES are a hydrogen bond acceptor (HBA), which can be a quaternary ammonium salt, and a hydrogen bond donor (HBD), such as alcohol or carboxylic acid. The resulting DES has a much lower melting point than that of the individual components. There are three classes of DESs: amide compounds (based on metal halide/imidazolium salt systems), thiocyanate/amide compounds (the combination of hydrated metal halides and choline chloride) and quaternary ammonium salts and HBDs (formed by choline chloride and several HBDs).^[^ ^136^ ^]^ Coelho et al. investigated the use of reline, a type III DES (choline chloride and urea), as solvent in ATRP. The polymerization of MA was performed by SARA ATRP with Me_6_TREN as ligand, ethyl *α*‐bromoisobutyrate as initiator, and ethanol as a cosolvent. The polymer had *Đ* < 1.19 and the high chain‐end functionality was confirmed by chain extension. The catalytic complex could be successfully recovered and used in two subsequent polymerizations.^[^ ^137^ ^]^ In another work, 100% reline was used as solvent for SARA ATRP of HEA, HEMA and (3‐acrylamidopropyl) trimethylammonium chloride (AMPTMA). The DES under study proved to be suitable for the use of different supplemental activators and reducing agents (Na_2_S_2_O_4_ and Cu^0^), demonstrating the versatility of the system.^[^ ^138^ ^]^ Iron‐mediated ATRP of MMA was performed using several pure DESs as both solvent and ligand at 60 °C. It was shown that the presence of a base (Na_2_CO_3_) decreased the polymerization time (from 4.5 to 1.5 h). However, it also had a negative effect on the polymerization control, leading to polymers with *Đ* > 1.50.^[^ ^139^ ^]^ 1,3‐Dimethyl‐2‐imidazolidinone (DMI) was explored as both ligand and solvent for the iron‐catalyzed AGET ATRP of MMA using several alcohols as reducing agents (Figures 1 and **11**).^[^ ^140^ ^]^

#### 4.5.5 Supercritical Carbon Dioxide

Another alternative solvent employed in the synthesis of polymers is supercritical CO_2_ due to its low cost, abundance, low toxicity, non‐flammability, and tunable solvent properties.^[^ ^141^ ^]^ ATRP was successfully performed in scCO_2_ using fluorinated (meth)acrylates for preparing block copolymers.^[^ ^142^ ^]^ Further studies used fluorinated macromonomers as both ligands and stabilizers for ATRP in scCO_2_, leading to polymers with predictable MW and low dispersity.^[^ ^143^ ^]^ Polymerization in scCO_2_ was also carried out by combining alkyne–azide Huisgen's cycloaddition and dispersion ATRP in a one‐pot process.^[^ ^144^ ^]^

### 4.6 P6: Design for Energy Efficiency

The major improvement in the energy efficiency of ATRP processes was achieved with the development of activator regeneration methods that can operate at room temperature, minimizing the energy demand of polymerizations. In contrast, FRP and conventional RDRPs, including “normal” ATRP and RAFT polymerization, typically rely on thermal initiation, requiring high temperature to i) decompose peroxides or diazo‐initiators and ii) drag the system to high conversion and/or to accelerate the process.^[^ ^18^, ^38^ ^]^ Alternative initiation methods and external stimuli have emerged as critical tools for energy‐efficient polymerizations.

In addition, the energy efficiency of polymerization processes can be highly improved by using continuous flow reactors, as well as by automatizing the operations and developing high‐throughput synthesis systems. Compared to batch reactors, continuous flow reactors offer improved productivity and reproducibility because the large surface area to volume ratio of tubular reactors provides efficient heat transfer, thus being convenient for polymerizations due to their exothermic nature. The facile modulation of synthetic conditions in flow reactors favors the development of high‐throughput polymer synthesis, which enables to rapidly optimize reaction parameters under highly reproducible conditions, and accelerate the catalytic system design.^[^ ^145^ ^]^ Finally, recent advances in computer science, robotics, and artificial intelligence are contributing to increase the efficiency of polymer research, by offering tools to model and automate reactions and accelerate innovation.

#### 4.6.1 Activator Regeneration‐Based ATRP Methods

From a kinetic point of view, ATRP with activator regeneration is fundamentally distinct from traditional ATRP.^[^ ^99^, ^146^ ^]^ The latter requires higher *K*_ATRP_ and/or higher concentration of Cu^I^/L to increase the polymerization rate (*R*_p_), according to Equation 1:

(1) $$R_{p}=k_{p}\left[M\right]\left[{P_{n}}^{•}\right]=k_{p}\;\left[M\right]\frac{K_{\mathrm{ATRP}}\left[{\mathrm{Cu}}^{I}/L\right]\left[P_{n}X\right]}{\left[\mathrm{XC}u^{\mathrm{II}}/L\right]}$$

Consequently, the polymerization rate was predominantly increased by enhancing the temperature (i.e., increasing *k*_p_ and *K*_ATRP_), since the catalyst loading was limited by its solubility.^[^ ^18a,c^ ^]^ More active catalysts were generally unfavorable for classic ATRP, as they could result in the generation of a large number of radicals at the beginning of the polymerization, hampering the initiation efficiency. Conversely, in ATRP with activator regeneration, the radical concentration, and thus *R*_p_ is proportional to the square root of the rate of activator regeneration (Equation 2). Therefore, the polymerization rate can be enhanced by increasing the rate of Cu^I^/L regeneration, which can be achieved by tuning the regenerating agent nature and loading, and the conditions.

In ICAR ATRP (Equation 2.1), the polymerization rate can be increased to some extent by increasing the concentration of radical thermal initiator or selecting an initiator with a higher decomposition rate constant (*k*_dc_) and efficiency (*f*). Faster ARGET ATRP is achieved by increasing the amount of reducing agent (Equation 2.2) or its reducing power, whereas in SARA ATRP a higher ratio of the surface of Cu wire to a reaction volume (*S*/*V*, Equation 2.3) results in increased polymerization rate.

(2) $$R_{p}=k_{p}\;\left[M\right]\;\left[{P_{n}}^{•}\right]=k_{p}\;\left[M\right]\sqrt{\frac{R_{{\mathrm{Cu}}^{I}/L\;\left(\mathrm{re}\right)\mathrm{generation}}}{k_{t}}}$$

(2.1) $$\mathrm{ICAR}\;\mathrm{ATRP}:\;\left[{P_{n}}^{•}\right]=\sqrt{\frac{fk_{\mathrm{dc}}\left[I_{2}\right]}{k_{t}}}$$

(2.2) $$\begin{array}{c} \mathrm{ARGET}\;\mathrm{ATRP}:\;\left[{P_{n}}^{•}\right]\;=\sqrt{\frac{k_{\mathrm{red}}\left[\mathrm{reducing}\;\mathrm{agent}\right]\left[X-{\mathrm{Cu}}^{\mathrm{II}}/L\right]}{k_{t}}} \end{array}$$

(2.3) $$\begin{array}{c} \mathrm{SARA}\;\mathrm{ATRP}:\;\left[{P_{n}}^{•}\right]=\sqrt{\frac{k_{a0}\left(S/V\right)\left[\mathrm{RX}\right]+k_{\mathrm{comp}}\left(S/V\right)\left[X-{\mathrm{Cu}}^{\mathrm{II}}/L\right]}{k_{t}}} \end{array}$$

Well‐controlled ARGET ATRP at room temperature has been reported for (meth)acrylates and acrylamides, in organic solvents or aqueous media, through a variety of reducing agents, including ascorbic acid (AA), Ag^0^, thiourea dioxide (TDO), as well as monomers containing a tertiary amine group.^[^ ^114^, ^147^ ^]^ AA and TDO emerge as green and inexpensive reducing agents, whereas Ag^0^ has the advantage of limited side reactions as its oxidation generates insoluble AgBr and enables room temperature Fe‐catalyzed ARGET ATRP.^[^ ^148^ ^]^ SARA ATRP of various monomers, including (meth)acrylates, acrylamides, and acrylonitrile was performed at room temperature, generally employing a Cu^0^ wire as a reducing agent and supplemental activator, but also Fe^0^, inorganic sulfites, *N,N,N′,N*′‐tetramethyl guanidine and polymer grafted liquid metal nanoparticles.^[^ ^23^, ^149^ ^]^ SARA ATRP benefits from simple setup, generally providing nearly quantitative conversion in a relatively short time, with high chain‐end functionality, allowing for one‐pot synthesis of block copolymers.^[^ ^150^ ^]^ The addition of small percentages of water (10–20 vol%) could result in >1.5 times faster SARA ATRPs, thanks to the enhanced catalytic activity.^[^ ^151^ ^]^ Mixtures of IL, glycol, and water enabled “flash” SARA ATRP of methyl acrylate, reaching >90% conversion in *ca*. 10 min at room temperature.^[^ ^152^ ^]^

Activator regeneration can be also achieved through external stimuli, such as light, electric current/potential and ultrasounds.^[^ ^27^ ^]^ *Photo*ATRP provides temporal and spatial control over polymerizations and can employ conventional Cu and Fe‐based ATRP catalysts in the presence of electron donors, Ir and Cu‐based photocatalysts, and organocatalysts, under UV or visible light irradiation.^[^ ^18^, ^27^ ^]^ Alternatively, dual catalytic systems were developed where a photocatalyst activated a Cu catalyst by photoinduced energy/electron transfer. This strategy allowed to perform *photo*ATRP under green and red light irradiation, by using a conjugated microporous polymer containing a phenothiazine (PTZ‐CMP) motif as a heterogeneous photosensitizer for the (re)generation of Cu^I^/L via photoinduced energy/electron transfer.^[^ ^153^ ^]^ PTZ‐CMP was separated from the final solution by centrifugation and effectively reused multiple times, thus allowing for using green or red light irradiation with conventional Cu complexes (cf. P9: Catalysis). NIR light was recently used for the *photo*ATRP of monomers comprising UV blocking moieties, as well as by using upconversion nanoparticles as recoverable and reusable internal light converter.^[^ ^154^ ^]^ The rate of *photo*ATRP is generally tuned by varying the irradiation intensity and wavelength, or the concentration of the electron donor. Cu‐mediated *photo*ATRP was performed with very low catalyst loadings in various media, including benign (mini)emulsion systems and water,^[^ ^69^, ^124^ ^]^ and in open‐to‐air systems via PICAR ATRP^[^ ^33c^ ^]^ (cf. P3: Less hazardous synthesis).

Electrochemically mediated ATRP uses an applied potential or current to (re)generate Cu^I^/L, thus no byproducts are generated and the polymerization rate is tuned by changing the applied potential or current intensity.^[^ ^25^, ^155^ ^]^ *e*ATRP has been performed at room temperature for (meth)acrylates, acrylonitrile, and acrylamides, in organic solvents, water, miniemulsion, and ionic liquids.^[^ ^119^, ^155^, ^156^ ^]^ The reaction setup is highly versatile and can be as simple as a current generator and two electrodes. The working electrode can be made of virtually any material, provided that it does not interfere with the reaction, and it should have a high surface area to promote fast electron transfer.^[^ ^157^ ^]^ The wall of a stainless reactor can serve as a working electrode itself, evidencing the scalability of this technique.^[^ ^158^ ^]^ Furthermore, a sacrificial counter electrode can be used, generally made of Al, which is immersed in the same solution as the working electrode, in a largely simplified setup.^[^ ^159^ ^]^ Importantly, *e*ATRP is a good candidate to harness the electricity coming from renewable resources and fits into the targeted electrification of the chemical industry.

*Mechano*ATRP is based on using piezoelectric materials and ultrasound to reduce X‐Cu^II^/L.^[^ ^26a^ ^]^ The polymerization rate depends on the nature and loading of the piezoelectric material and the excess of ligand L, which is oxidized in the process.^[^ ^160^ ^]^ In aqueous media, ultrasound can be used to generate hydroxyl radicals in the absence of piezoelectrics, thus *sono*ATRP is a simpler and more environmentally friendly system.^[^ ^26b^ ^]^ Despite radicals are continuously generated, their concentration is low so that block copolymers and temporal control can still be achieved. The polymerization rate is tuned by changing the sonication frequency.^[^ ^161^ ^]^

Combined with increasingly more active catalysts, these innovative initiating systems contributed to greener ATRP.^[^ ^99b^ ^]^ The use of low catalyst loading diminishes the energy required to run polymerizations: for instance, the current needed to drive an *e*ATRP to high conversion is proportional to the initial concentration of X‐Cu^II^/L and to the number of termination reactions that build up X‐Cu^II^/L.^[^ ^155^ ^]^ Since terminations in ATRP generally account for less than 10% of chains, the energy consumption is primarily dictated by the catalyst loading. Moreover, more active catalysts typically drive faster polymerizations. However, an increase in the ATRP activity corresponds to a more negative reduction potential of the X‐Cu^II^/L complex.^[^ ^99b^ ^]^ Highly active catalysts are therefore more difficult to reduce, and strong reducing agents or conditions might be needed to obtain reasonably rapid polymerizations.

#### 4.6.2 Continuous Flow Reactors

**Figure 12:**
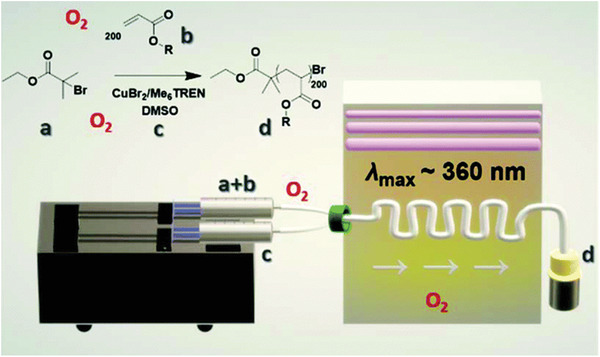
Schematic of photoinduced ATRP in continuous flow without external deoxygenation. Reproduced with permission.^[^ ^168^ ^]^ Copyright 2019, Royal Society of Chemistry.

The first implementations of normal ATRP in flow reactors required high temperatures and catalyst loadings, nonetheless demonstrating good control, facile parameter modulation, and synthesis of block copolymers.^[^ ^162^ ^]^ The advent of activator regeneration techniques enabled to overcome initial limitations. For example, copper tubular reactors in which the walls serve as a supplemental activator and reducing agent were designed and optimized to achieve high conversion at relatively low ligand loading and room temperature.^[^ ^163^ ^]^ This concept was successively translated to Cu^0^ mediated ATRP in a quartz crystal microbalance (QCM), which further allowed for in situ monitoring of the polymerization (cf. P11: Real‐time analysis).^[^ ^164^ ^]^ A similar approach was adopted for Fe‐mediated ATRP in flow, using iron tubing and Fe salt with no additional ligands.^[^ ^165^ ^]^ Photopolymerizations are well suited for continuous flow reactors as the tubular setup increases the light efficiency, avoiding nonuniform penetration and gradients that are common for large‐volume photopolymerizations in batch.^[^ ^166^ ^]^ *Photo*ATRP in continuous flow has been performed with Cu and Ir catalysts, as well as organocatalysts.^[^ ^167^ ^]^ Faster reactions and improved control were observed compared to similar batch polymerizations, owing to the efficient light irradiation and lower oxygen permeability. Cu‐catalyzed *photo*ATRP in flow with no external deoxygenation (**Figure** **12**) has been recently achieved by carefully dosing the catalyst and excess ligand.^[^ ^168^ ^]^ Importantly, continuous ATRPs exhibited comparable monomer and architecture scope, and chain‐end fidelity to batch processes. The efficient irradiation achieved in flow systems resulted in more effective photoexcitation of organocatalysts for ATRP, thus faster activation, which in turn increased the deactivator concentration, improving the control compared to batch processes.^[^ ^169^ ^]^

#### 4.6.3 High‐Throughput Systems

**Figure 13:**
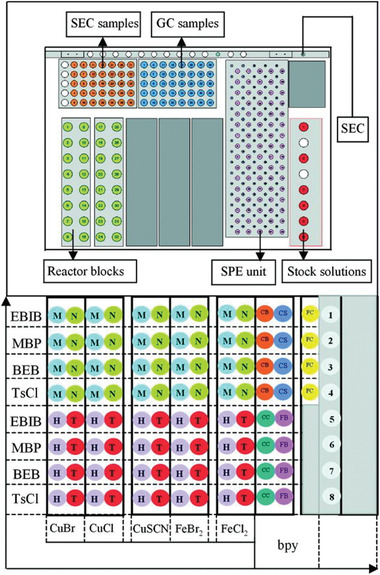
Schematic setup of an automated synthesizer and combinations of metal salts, initiators, and ligands for ATRP. Reproduced with permission.^[^ ^170^ ^]^ Copyright 2004, Wiley.

High‐throughput studies in ATRP are facilitated by the relatively low cost of the catalysts and the possibility to work at room temperature with activator regeneration. These studies were conducted in automated synthesizers (**Figure** **13**), generally connected to an online size exclusion chromatography (SEC) instrument and other online or offline characterization machines.^[^ ^170^ ^]^

**Figure 14:**
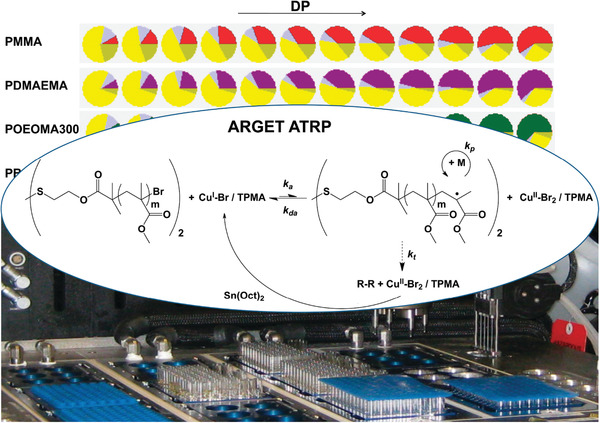
Setup and scheme of high‐throughput ARGET ATRP. Reproduced with permission.^[^ ^173^ ^]^ Copyright 2012, American Chemical Society.

Parallel ATRPs with Cu and Fe‐based catalysts accelerated the understanding of structure–activity relationships, and enabled the rapid synthesis of (co)polymer libraries, even at room temperature, via SARA ATRP.^[^ ^170^, ^171^ ^]^ The removal of Cu catalysts was effectively integrated into high‐throughput systems,^[^ ^172^ ^]^ and the modest oxygen tolerance of some ATRP techniques with activator regeneration allowed for performing automated ARGET ATRP in sealed nondegassed vials (**Figure** **14**). The optimized conditions applied to a vast monomer range were used to synthesize thiol terminated polymers as precursors for bioconjugates.^[^ ^173^ ^]^

High‐throughput and automated methods are particularly relevant for the synthesis of biohybrids and biosensors. A DNA synthesizer was used for the automated preparation of homopolymers and block copolymers, and DNA–polymer hybrids via *photo*ATRP under UV light without deoxygenation.^[^ ^174^ ^]^ The use of blue light irradiation and enzymatic‐degassing enabled high‐throughput ATRP in completely open‐air well plates.^[^ ^69^ ^]^

#### 4.6.4 Application of Computation‐Based Methods

Modeling of ATRP using the Predici software^[^ ^175^ ^]^ and Monte Carlo method is increasingly used to distinguish reaction mechanisms, calculate reaction parameters, as well as predict reaction outcomes and guide conditions selection.^[^ ^176^ ^]^ Simulations of ICAR and ARGET ATRP helped optimize the loadings of the radical initiator or reducing agent, and ATRP catalyst, in order to achieve fast and controlled polymerizations.^[^ ^177^ ^]^ Modeling of ATRP in the presence of metallic Cu contributed to determining the role of Cu^0^ as a supplemental activator and reducing agent, confirming the SARA ATRP mechanism.^[^ ^178^ ^]^ Recently, a two‐compartment Monte Carlo model for *e*ATRP was proposed to effectively take into account the heterogeneous nature of the system, where the catalyst needs to diffuse toward and away from the electrode.^[^ ^179^ ^]^ Simulation of these systems provided conditions to balance polymerization rate and control, even attempting to push the limit of ATRP toward very fast polymerizations with preserved chain‐end functionality.^[^ ^180^ ^]^ Moreover, simulation models including side reactions (e.g., formation of organometallic intermediates) enabled to define conditions that improved the selectivity of ATRP catalysts.^[^ ^50^, ^181^ ^]^ Finally, simulations were applied to guide the synthesis of block and gradient copolymers, in batch, semibatch, and continuous reactors, and to identify the effect of catalyst loading on the synthesis of polymer brushes grafted from SiO_2_ nanoparticles.^[^ ^163^, ^182^ ^]^ Reinforcement learning has been applied to ATRP in order to control the shape of polymer molecular weight distribution.^[^ ^183^ ^]^ Neural network architectures were incorporated in a controller that was trained to guide the polymerization toward a target MW distribution, including bimodal distributions, by adding reagents at multiple points during the reaction. This promising approach has the potential to simultaneously target different properties and to transfer the controller to the laboratory.

### 4.7 P7: Use of Renewable Feedstocks

The seventh principle of green chemistry encourages the synthesis of chemicals from raw materials and/or renewable feedstocks whenever it is technologically and economically viable. Biobased plastics are necessary for a sustainable plastic future as they should: i) mitigate the climate and economic changes associated with depletion of fossil fuels, ii) exhibit minimal toxicity to humans and the environment (cf: P3: Less hazardous synthesis; cf. P4: Design benign chemicals), and iii) present degradation pathways, thus favoring the transition toward a zero‐waste economy (cf. P10: Design for degradation).^[^ ^184^ ^]^ To date, ATRP has been mainly used to generate new macromolecular architectures from oxygen‐containing biomasses, which are more susceptible to organic transformations into (meth)acrylate monomers.^[^ ^185^ ^]^

#### 4.7.1 Carboxylic Acid‐Based Monomers and Their Derivatives

**Figure 15:**
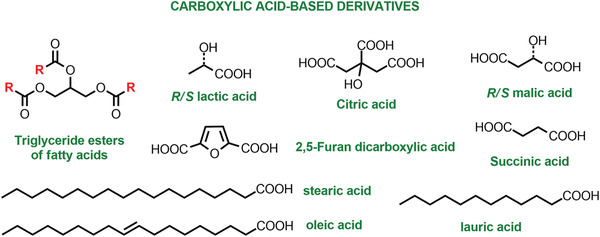
Examples of carboxylic acid‐based monomers and their derivatives relevant for ATRP.

The US Department of Energy identified 30 high‐value biobased compounds, including C_3_–C_6_ carboxylic acids such as lactic, malic, succinic, levulinic, citric acids, and 2,5‐furan dicarboxylic acid (**Figure** **15**).^[^ ^186^ ^]^ Great attention has been devoted to lactic acid, which is susceptible to hydrolysis, thus can serve as a substrate to form PLA‐based copolymers via ATRP to tune the mechanochemical properties and degradation of PLA. PMMA‐graft‐PLA graft copolymers were synthesized via a grafting from approach, using methacrylic PLA macromonomers.^[^ ^187^ ^]^ Moreover, PLA was functionalized with ATRP initiating sites, followed by the formation of PMMA brushes and subsequent functionalization with gelatin for improved cell‐adhesion properties.^[^ ^188^ ^]^ PLA‐macroinitiators with thiol linkages were employed to prepare block copolymers with styrene, acrylate, or methacrylate segments in a two‐step ROP and ATRP process.^[^ ^189^ ^]^ Such materials exhibited good processability and possible degradation pathways (cf. P10: Design for degradation).

Raw vegetable oils can be subjected to hydrolysis or transesterification towards glycerol and fatty acids (FA) and serve as substrates for ATRP. The application of ATRP to FA‐based monomers was recently summarized.^[^ ^190^ ^]^ Long‐alkyl‐chain additives (as quaternary ammonium salts, e.g., Aliquat 336) or ligand modifications were generally required in ATRP of FA‐based monomers to enhance monomer/polymer solubilities. These studies mainly focused on the more common stearyl and lauryl (meth)acrylates, although other methacrylates were explored with different lengths of the side chains, including C_10_, C_14,_ and C_16_.^[^ ^191^ ^]^ Furthermore, metal‐free *photo*ATRP was efficiently utilized as a green strategy to synthesize well‐defined polymers from biomass using soybean oil as a precursor,^[^ ^192^ ^]^ and to graft lauryl methacrylate from cellulose.^[^ ^193^ ^]^ It is however fundamental to consider the seasonal availability of several biobased monomers and the heterogeneity of their chemical composition, in addition to the indispensable, relatively costly extractions.

#### 4.7.2 Lignocellulosic Biomass (LCB)

**Figure 16:**
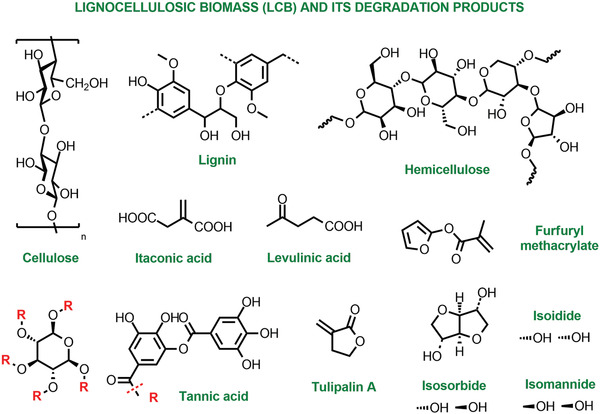
Examples of lignocellulosic biomass and its degradation products relevant for ATRP.

LCB is the largest biomass source on Earth and comprises natural polymeric materials in the form of cellulose, hemicellulose, and lignin (**Figure** **16**).^[^ ^194^ ^]^ LCB either in pristine or degraded forms can be successfully utilized as renewable feedstocks for a broad variety of catalytic processes, which makes LCB among the most promising source for sustainable materials. Regarding cellulose‐based materials, ATRP has been efficiently used to form various macromolecular architectures, using strategies to improve the solubility and processability of the pristine cellulose polymer.^[^ ^195^ ^]^ The efficacy of low‐ppm catalyst ATRP techniques, and the facile tuning of the properties of copolymers prepared by grafting from cellulose‐based macroinitiators has been recently reviewed.^[^ ^196^ ^]^ These well‐defined materials find promising applications as bioassays. In addition, cellulose‐derived polymers can display tunable degradation (cf. P10: Design for degradation), thus being key materials in a circular plastic economy. Lignin is the second most abundant biomass polymeric material, after cellulose, and it is an industrial byproduct, predominantly used as an energy source.^[^ ^194^ ^]^ ATRP was used to prepare lignin‐derived composite materials, mostly thermoplastics,^[^ ^196^, ^197^ ^]^ albeit lignin is less studied than cellulose. ARGET ATRP was successfully applied for the construction of poly(2‐dimethylaminoethyl methacrylate) (PDMAEMA) grafted from lignin nanofiber mats,^[^ ^198^ ^]^ and to graft copolymers of MMA and styrene from lignin macroinitiators via AGET ATRP with an iron‐based catalyst.

#### 4.7.3 Monosaccharides and Their Degradation Products

Monosaccharides obtained from the degradation of cellulose were utilized as renewable feedstocks, with tannic acid, isosorbide, isomannide, and isoidide as examples (Figure 16).^[^ ^199^ ^]^ The presence of diol functionality renders them suitable for chemical derivatization for making mono‐ or bis‐(meth)acrylate monomers to prepare soluble polymeric or crosslinked materials, respectively. Chain‐growth polymerization of isosorbide was predominantly studied by RAFT polymerization.^[^ ^200^ ^]^ However, the synthesis of miktoarm star copolymers from a soybean oil‐derived macroinitiator was achieved by sequential ATRP of an isosorbide‐based monomer and ROP of *ε*‐caprolactone.^[^ ^201^ ^]^ The application of renewable tannic acid (TA) derivatives will be covered later (cf. P10: Design for degradation), due to the degradable character of the TA moiety.

The degradation of C_5_ and C_6_ monosaccharides leads to the formation of the first‐generation furan derivatives, i.e., furfural and its hydroxymethyl derivatives, which are chemical commodities widely explored for polycondensation reactions.^[^ ^202^ ^]^ In order to prepare furfural‐based polymers via ATRP, the preferred pathways involve the reduction of furfural to the corresponding alcohol and subsequent derivatization to form FMA. Normal ATRP of FMA yielded homopolymers with predetermined MW and moderately low dispersity (*Đ* = 1.3).^[^ ^203^ ^]^ PFMA was easily processable and employed as a reversible thermoset by exploiting reversible Diels‐Alder chemistries (cf. P10: Design for degradation). PFMA was also prepared by performing metal‐free *photo*ATRP of FMA, with 10‐phenylphenothiazine as organocatalyst.^[^ ^192^ ^]^ Furthermore, grafting from or grafting onto ATRP methods were recently adopted to form cellulose‐based brushes with furfural‐derived side chains.^[^ ^88^, ^193^ ^]^ Itaconic acid (IA) is a biobased dicarboxylic acid monomer and a precursor of 3‐methyltetrahydrofuran.^[^ ^204^ ^]^ SI‐ATRP was employed to grow PIA brushes from gold surfaces in aqueous solutions, which were patterned via self‐assembled monolayers (SAMs) of 11‐mercapto‐undecanol functionalized with 4‐(chloromethyl) benzoyl chloride initiator.^[^ ^205^ ^]^ *N*‐phenylitaconimide was polymerized by ICAR ATRP catalyzed by FeBr_3_, then the polymer was used as a macroinitiator to form a copolymer with styrene,^[^ ^206^ ^]^ and homopolymers of dimethyl itaconate^[^ ^207^ ^]^ and their copolymers with acrylonitrile by ARGET ATRP.^[^ ^208^ ^]^ Thus, biomass‐derived monomers were effectively paired with greener synthetic conditions, such as low catalyst loadings and benign Fe‐based catalysts.

IA, together with levulinic acid (LA),^[^ ^209^ ^]^ are precursors of five‐membered *γ*‐butyrolactones with structural similarity to methacrylates. Functional polymeric materials obtained from  *α*‐unsaturated *γ*‐butyrolactones could replace some commodity polymers due to their attractive properties: high glass transition temperature (*T*_g_), heat‐, solvent‐ and scratch resistance.^[^ ^210^ ^]^ The simplest representative of this group, *α*‐methylene‐*γ*‐butyrolactone (MBL or Tulipalin A, Figure 16), derived from tulips, was polymerized by ATRP to prepare polymers with predetermined MW and low dispersity.^[^ ^211^ ^]^ TPEs were obtained by synthesizing triblock^[^ ^212^ ^]^ and star‐shaped^[^ ^213^ ^]^ copolymers of MBL and *n*BA, with excellent mechanical and thermal properties. ATRP was combined with ring‐opening transesterification polymerization (ROTEP) to prepare bioderived ABA triblock copolymers from menthide and MBL,^[^ ^214^ ^]^ and high‐density PMBL brushes grown on a silicon substrate via SI‐ATRP.^[^ ^215^ ^]^ Recently, Tulipalin A‐based polymers were prepared through an environmentally benign *photo*ATRP process with low‐ppm content of Cu catalyst and in oxygen‐tolerant conditions.^[^ ^216^ ^]^

#### 4.7.4 Terpenes and Terpenoids

**Figure 17:**
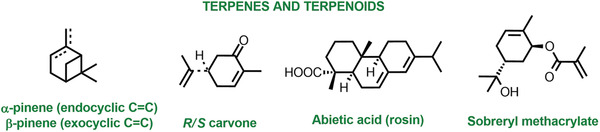
Examples of terpenes and terpenoids used in ATRP.

Another important group of renewable materials are wood‐derived species, including extractable terpenes and terpenoids, as well as the nonvolatile pine resin termed rosin (**Figure** **17**).^[^ ^217^ ^]^ The carbonyl or carboxylic acid functionalities in terpenoids (e.g., carvone) or rosin acids (e.g., dehydroabietic acid), and their chemical transformations (e.g., in terpenes) were used to prepare monomers for ATRP, generating a poly(*β*‐pinene)‐*graft*‐polystyrene copolymer from a semibrominated poly(*β*‐pinene) macroinitiator,^[^ ^218^ ^]^ and to grow rosin‐derived polymers from cellulose through the grafting from approach.^[^ ^219^ ^]^ *α*‐Pinene was transformed to sobrerol and subsequently to methacrylate and polymerized via FRP, RAFT, and normal ATRP.^[^ ^220^ ^]^ The obtained biorenewable polymers were crosslinked to form thin films via photoinduced thiol–ene click chemistry or by thermal curing with hexamethoxymethylmelamine.

### 4.8 P8: Reduce Derivatives

Some protecting groups in monomers used for ATRP are not necessarily due to the tolerance of ATRP systems to a broad range of functionalities. For instance, poly(acrylic acid) and poly(methacrylic acid) were recently prepared directly from acidic monomers by ATRP at low pH (cf. P2: Atom economy).^[^ ^52^ ^]^ Previously they were synthesized by hydrolysis of poly(*tert*‐butyl acrylate) and poly(*tert*‐butyl methacrylate), respectively. Some monomers, initiators, or catalysts can have a dual role in the ATRP equilibrium, thus allowing for reducing the number of reagents and simplifying the experimental setup.

#### 4.8.1 ATRP of MMA Catalyzed by Fe Halide Salts

**Figure 18:**
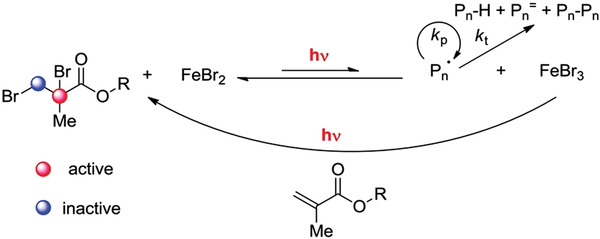
Scheme of *photo*ATRP of MMA in the presence of FeBr_3_ as the only required reagents. Reproduced with permission.^[^ ^223^ ^]^ Copyright 2017, Wiley.

Iron‐based ATRP can be performed using Fe halides as catalysts without the need for additional ligands and/or initiators.^[^ ^51^ ^]^ FeBr_2_ was used as a catalyst for ATRP of methacrylates in the presence of polar solvents as ligands able to solubilize the catalyst and regulate its activity.^[^ ^221^ ^]^ Fe‐based *photo*ATRP of methacrylates was carried out with air‐stable FeBr_3_ and RX initiators, without additional ligands, reducing agents, or thermal radical initiators.^[^ ^222^ ^]^ Indeed, ATRP of MMA under UV light irradiation using FeBr_3_, EBPA, and CH_3_CN acting as solvent and ligand provided well‐defined polymers with high chain‐end functionality. Temporal control was demonstrated by switching the light on/off. In this process, the FeBr_2_ activator was formed by photoreduction of FeBr_3_, generating 2,3‐dibromoisobutyrate. The latter could act as ATRP initiator, and therefore a further simplification was achieved using only monomer and FeBr_3_, in the absence of any alkyl halides.^[^ ^223^ ^]^ In this system, FeBr_3_ played the role of deactivator, source of FeBr_2_ activator, and initiator (**Figure** **18**). Similarly, *photo*ATRP of MMA was carried out using FeCl_3_ as catalyst in the presence of tris(4‐methoxyphenyl)phosphine as ligand, but in the absence of RX initiator that was replaced by the in situ forming 2,3‐dichloroisobutyrate.^[^ ^224^ ^]^ The system was later extended to a ppm level of catalyst loading with visible light irradiation.^[^ ^225^ ^]^

#### 4.8.2 Monomers with a Dual Role

**Figure 19:**
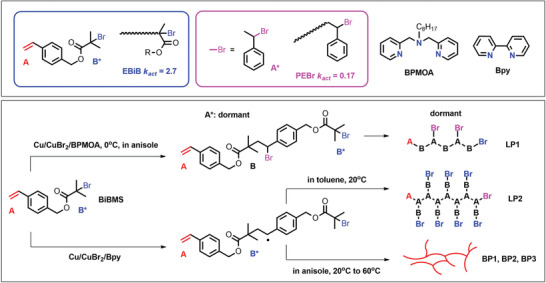
Control of polymer topology in the SCVP ATRP of *p*‐(2‐bromoisobutyroylmethyl)styrene (BiBMS). Reproduced with permission.^[^ ^231^ ^]^ Copyright 2010, American Chemical Society.

The DMAEMA monomer contains a tertiary amine group that could act as intrinsic reducing agent for the regeneration of the ATRP activator and to compensate for the presence of a limited amount of oxygen.^[^ ^147c^ ^]^ Therefore, in ATRP of DMAEMA, external reducing agents are not needed. Moreover, ATRP was conducted using inimers (AB*), i.e., vinyl monomers containing both a double bond (A) and an initiating site (B*). Inimers can undergo a polymerization via the vinyl function, initiate a new chain from the halogen site or generate a branch after incorporation. Thus, inimers have a dual role of both monomers and initiators. Inimers were first introduced in cationic self‐condensing vinyl polymerization (SCVP).^[^ ^226^ ^]^ ATRP provided good control of polymer architectures and of the chain length between two branching points, by copolymerization of a monomer with an inimer and adjusting their molar ratio.^[^ ^227^ ^]^ The first SCVP by ATRP was studied using *p*‐(chloromethyl)styrene (CMS) in the presence of Cu^I^/bpy.^[^ ^228^ ^]^ ATRP SCVP was employed with acrylates,^[^ ^227^, ^229^ ^]^ methacrylates,^[^ ^230^ ^]^ and styrenic^[^ ^231^ ^]^ monomers containing either alkyl chloride or alkyl bromide initiating sites (**Figure** **19**).

**Figure 20:**
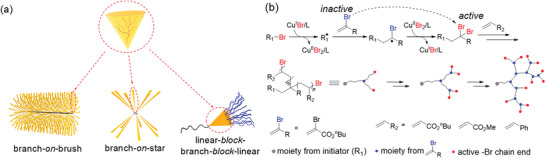
a) Hierarchically branched polymers constructed from branched‐polymer building blocks. b) Simplified mechanism. Reproduced with permission.^[^ ^233^ ^]^ Copyright 2018, American Chemical Society.

The structure of polymers obtained by SCVP ATRP depends on the competition between reactions of propagation and deactivation. Propagating radicals formed by activation of alkyl halides can react with a new inimer and form linear structures, or be deactivated enabling activation of a different alkyl halide site, forming branched structures.^[^ ^232^ ^]^ A new method was recently developed to prepare hyperbranched polymers with hierarchically branched architecture (**Figure** **20**). This method employed inibramers (initiator–branching agents–monomers), of, i.e., *α*‐haloalkenes (e.g., *n*‐butyl *α*‐bromoacrylate (BBA)) used in ATRP copolymerization with conventional monomers. C—Br bond in  *α*‐haloalkenes has much higher bond dissociation energy than in conventional initiators and branching occurred only after BBA was incorporated into a polymer chain forming tertiary alkyl halide, additionally activated by the ester group.^[^ ^233^ ^]^

### 4.9 P9: Catalysis (vs Stoichiometric)

Original ATRP systems required a nearly stoichiometric amount of Cu complexes relative to RX initiators (*ca*. 1 mol% or thousands of ppm vs monomer) to overcome the effects of radical termination and the relatively low activity of traditionally employed catalysts.^[^ ^38b^ ^]^ However, the development of new ligands that form complexes with much higher activity toward alkyl halides and the invention of techniques with continuous regeneration of the activator allowed for decreasing of the catalyst loading to below 10 ppm (0.001 mol%). Nowadays, the use of catalysts with activity billion times higher than that of the first catalyst used in ATRP enables to perform polymerizations at room temperature, resulting in colorless products that do not require further purification for many applications.^[^ ^234^ ^]^ This represents a substantial advantage of ATRP over, for example, RAFT polymerization where the stoichiometric amount of a RAFT agent results in colored polymers, especially for low MW polymers. In addition, several methods were reported to further remove traces of the transition metal catalyst,^[^ ^235^ ^]^ or to minimize contaminations by using heterogenous catalysts. Alternatively, more benign Fe‐based and organocatalysts were also employed in ATRP. Besides its activity, the selectivity of an ATRP catalyst affects the polymerization outcomes (cf. P2: Atom Economy).

#### 4.9.1 Removal of Cu Catalysts

Cu‐based ATRP catalysts were removed from the final mixtures by passing the polymer solution through adsorbent (silica or neutral alumina column),^[^ ^234^ ^]^ ion exchange resins,^[^ ^236^ ^]^ or clay,^[^ ^237^ ^]^ or by using an ionic liquid as polymerization solvent enabling catalyst removal and recycling.^[^ ^126^ ^]^ Alternatively, the catalyst was recycled upon precipitation into a nonsolvent,^[^ ^238^ ^]^ using precipitants bound to ligands. Another strategy consists of electrodeposition, whereby all copper complexes are reduced to metallic Cu that is deposited onto the electrode surface, and thus can be quantitatively removed from the solution.^[^ ^239^ ^]^

#### 4.9.2 Heterogeneous Catalysis

Heterogeneous catalysis could overcome the hurdles of homogeneous catalyst removal. Two approaches were mainly adopted toward heterogeneously catalyzed ATRP: i) physical or chemical immobilization of the ATRP catalyst on a solid support, and ii) use of liquid/liquid biphasic catalyst systems such as ionic liquids, fluorous and aqueous systems, and polymer‐bound catalysts.^[^ ^240^ ^]^ A reversibly supported catalyst, when the catalyst exchanges between the support and the solution, is also compatible with flow reactors.^[^ ^241^ ^]^ Typically the control in heterogeneous ATRP is limited by the decreased catalyst mobility, which can prevent effective deactivation of rapidly diffusing radicals, resulting in polymers with relatively high dispersity.^[^ ^242^ ^]^ This drawback can be partially overcome by improving the catalyst mobility through a spacer between the support and the catalyst.^[^ ^243^ ^]^

On the other hand, biphasic systems are often preferred, as they offer highly active catalytic performance and facile catalyst removal. Moreover, aqueous/organic biphasic systems are used in large‐scale applications, since they use economical and environmentally friendly water, as well as highly efficient water‐soluble catalysts. Biphasic catalyst systems were further improved through the development of thermoregulated phase‐transfer catalysts (TRPTCs),^[^ ^244^ ^]^ thermoregulated phase separable catalysts (TPSCs),^[^ ^245^ ^]^ or water‐induced phase separable catalysts (WPSCs).^[^ ^246^ ^]^ TRPTCs exploit the insolubility at low temperature of employed catalysts, which can include fluorinated ligands^[^ ^247^ ^]^ or PEG‐supported pyridyl ligands.^[^ ^248^ ^]^ On the contrary, in TPSCs, the copper catalyst is initially in the ionic liquid phase, which is immiscible with the monomer at room temperature. Polymerization occurs upon increasing the temperature and making the mixture homogeneous. Cooling of the final solution results in the separation of the polymer from the catalyst/ionic liquid layer. In WPSC, *ca*. 10 vol% water is added to induce phase separation between the hydrophilic polymer and the solvents mixture containing the copper complex.

**Figure 21:**
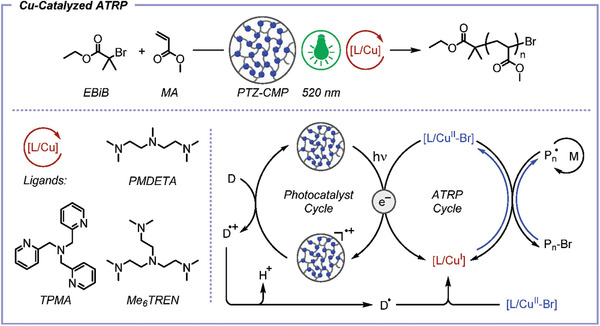
Photoinduced copper‐catalyzed ATRP in the presence of PTZ‐CMP as a heterogeneous photocatalyst used to generate the Cu^I^/L activator catalyst under green light irradiation in the presence of amine electron donors (*D*). Reproduced with permission.^[^ ^153^ ^]^ Copyright 2021, American Chemical Society.

Conjugated microporous polymers with phenothiazine photoactive cores (PTZ‐CMP) act as reusable heterogeneous photocatalysts (cf. P6: Design for energy efficiency).^[^ ^153^ ^]^ ATRP of methacrylate and acrylate monomers was performed under green and red light with high conversion and well‐controlled molecular weights (**Figure** **21**).

#### 4.9.3 Fe‐Catalyzed ATRP

Iron‐based catalysts have attracted a considerable degree of attention in ATRP because iron is more earth abundant, less expensive, and usually less toxic than copper.^[^ ^51^ ^]^ The first reports on Fe‐mediated ATRP of MMA and styrene investigated the relatively simple (NR_3_)_3_Fe^II^(Br)_2_ complex (R = butyl or octyl) with substituted bpy ligands, triphenylphosphines, trialkylphosphines, and trialkylphosphites.^[^ ^249^ ^]^ Complexes of Fe with diamine ligands are involved in polymerization through an ATRP mechanism or a catalytic chain‐transfer (CCT) mechanism, depending on the spin‐state of the resulting Fe^III^ species, where the high spin system (*S* = 5/2) promotes ATRP, and the intermediate spin (*S* = 3/2) promotes CCT.^[^ ^250^ ^]^ Overall, factors such as the spin state of the complex, the nature of the radical and the ligand scaffold determine the mechanism of polymerizations regulated by Fe‐based catalysts.^[^ ^251^ ^]^ Since iron halides are the most versatile Fe catalysts, they were extensively explored in *photo*ATRP, without the need of any additional additive or reducing agent (cf. P8: Reduce derivatives).^[^ ^222^ ^]^ Iron halides such as Fe^II^Br_2_ and Fe^III^Br_3_, in the presence of additional halides can generate anionic complexes like Fe^II^Br_4_^2−^, Fe^III^Br_4_^−^ or Fe^III^Br_5_^2−^,^[^ ^252^ ^]^ where Fe^II^(S)Br_3_^−^ (S = solvent) is the main activator and Fe^III^Br_4_^−^ is the main deactivator.^[^ ^253^ ^]^ Less polar solvents give faster activation, the opposite to what is observed for Cu‐based catalysts.

#### 4.9.4 Metal‐Free ATRP

**Figure 22:**
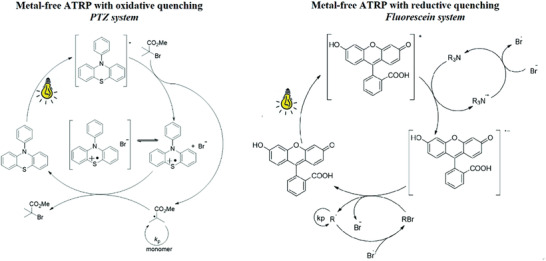
Mechanism for metal‐free ATRP with oxidative quenching (PTZ system) and reductive quenching (fluorescein system). Reproduced with permission.^[^ ^27^ ^]^ Copyright 2018, Royal Society of Chemistry.

Conventional transition metal catalysts in ATRP can be replaced with green organocatalysts (**Figure** **22**) in metal‐free/organo‐catalyzed ATRP (*o*ATRP).^[^ ^27^, ^254^ ^]^ The organic photoredox catalyst 10‐phenyl phenothiazine (Ph‐PTZ) was reported to successfully control the polymerization of MMA or pyridine‐based monomers under UV or LED irradiation at room temperature.^[^ ^254^, ^255^ ^]^ Ph‐PTZ, 10‐(4‐methoxyphenyl)‐phenothiazine (4‐MeOPh‐PTZ) and 10‐(1‐naphthalenyl)‐phenothiazine (Nap‐PTZ) were employed in the *o*ATRP of acrylonitrile obtaining good control over molecular weights and temporal control, but the polymers exhibited *Ð* > 1.40.^[^ ^256^ ^]^

The mechanism of ATRP was studied for the polymerization of MMA using several different photoredox catalysts.^[^ ^257^ ^]^ Their structure and reactivity for both the activation of (macro)alkyl halides and the deactivation of propagating radicals were evaluated by kinetic, electrochemical, photophysical, and theoretical computational studies. In metal‐free ATRP based on oxidative quenching, the photocatalyst enters its excited state via absorption of a photon of sufficient energy; then, the excited photocatalyst activates alkyl halides to generate radicals. Deactivation was proposed to proceed through termolecular associative electron transfer between a radical, a bromide anion, and the oxidized photocatalyst (radical cation), or by formation of an ion‐pair between the radical cation and a bromide anion, followed by reaction of the ion‐pair with a radical. Typical organic photocatalysts for ATRP include polycyclic aromatic hydrocarbons,^[^ ^258^ ^]^ phenothiazines,^[^ ^257^ ^]^ dihydrophenazines,^[^ ^259^ ^]^ phenoxazines,^[^ ^260^ ^]^ and carbazoles.^[^ ^261^ ^]^ In *o*ATRP both the singlet excited state photocatalyst (^1^PC*) and the triplet excited state photocatalyst (^3^PC*) can participate in the activation reaction. The ^1^PC* is more reducing than ^3^PC*, but the latter ensures better activation because it has a much longer excited‐state lifetime due to the ability to access a charge‐transfer (CT) triplet excited state, paramount to stabilize the ^3^PC* species.^[^ ^262^ ^]^ Besides CT, solvent polarity plays also significant effect on *o*ATRP. More polar solvents may destabilize the PC^•+^Br^−^ ion‐pair, hampering the deactivation efficiency. Proper design of catalyst in *o*ATRP resulted in increased control of polymerization (*Đ* < 1.10). *o*ATRP enabled to synthesize star copolymers through a core‐first approach^[^ ^263^ ^]^ and it was employed in a continuous flow synthesis.^[^ ^169a^ ^]^ Alkyl halide activation in *o*ATRP can also proceed via a reductive quenching pathway in the presence of electron donors using photocatalysts such as eosin Y, erythrosine B,^[^ ^264^ ^]^ and fluorescein.^[^ ^265^ ^]^ Contrary to the oxidative quenching pathway where the excited photocatalyst directly activates RX, in the reductive quenching pathway PC* reacts with the electron donor, such as triethylamine or PMDETA, generating a radical anion (PC^•−^) that activates RX.^[^ ^153^, ^266^ ^]^

#### 4.9.5 Enzymatic Catalysis

Enzymes are nontoxic, naturally sourced and biodegradable; they work under mild conditions (below 100 °C), exhibit stereo‐, regio‐, and substrate selectivity, generally present a high catalytic turnover and can be easily removed from reaction products.^[^ ^267^ ^]^ Biocatalytic ATRP (bioATRP) was implemented through the use of various metalloenzymes, such as HRP,^[^ ^268^ ^]^ hemoglobin (Hb),^[^ ^269^ ^]^ catalase (CBL), and laccase (LTV).^[^ ^270^ ^]^ Enzymatic catalysis is suitable for a wide range of monomers, due to the available pool of catalyst structures and their tolerance to pH variation. On the other hand, biocatalysts typically have high molecular weights and high sensitivity to reaction conditions, therefore synthetic analogs were developed with improved stability to broaden the scope of their applications.^[^ ^271^ ^]^ Hemin‐inspired catalysts that addressed these issues were developed and showed significantly improved performance in the preparation of well‐defined polymers.^[^ ^272^ ^]^ Bio‐inspired iron‐porphyrin‐based complexes were designed and successfully used as catalysts for ATRP of methacrylic acid.^[^ ^273^ ^]^

### 4.10 P10: Design for Degradation

This principle confers to building a circular plastic economy, a solution to the unacceptable growth of polymer waste,^[^ ^274^ ^]^ possible through introduction of cleavable and/or reversible chemical subunits to the polymeric material or utilization of depolymerization strategies. The radical nature of ATRP makes it suitable to utilize both methods. Note that some biobased polymers (cf. P7: Use of renewable feedstocks) are not necessarily biodegradable, and that such materials have been synthesized using both fossil feedstocks and renewable resources.

**Figure 23:**
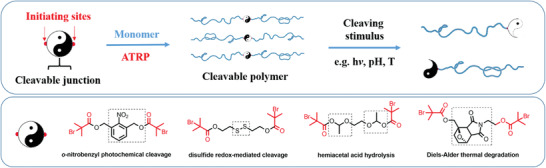
Examples of degradable chemistries in ATRP.

The role of ATRP to build (bio)degradable polymer materials is mostly through the use of cleavable monomers or initiators, with chemistries based on disulfides, acetals, Diels‐Alder or photocleavable chemistries (**Figure** **23**).^[^ ^38^, ^275^ ^]^

#### 4.10.1 Radical Ring‐Opening Polymerization (RROP)

**Figure 24:**
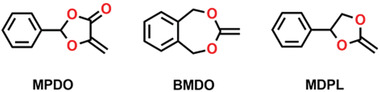
Monomers utilized for RROP polymerizations via ATRP.

Cyclic monomers with a radical acceptor (carbon–carbon double bond) and thermodynamically favored ring‐opening process can generate degradable polymeric architectures, with most prominent being cyclic ketene acetals (CKAs) and cyclic acrylates (**Figure** **24**).^[^ ^276^ ^]^

ATRP was applied to polymerize 5‐methylene‐2‐phenyl‐1,3‐dioxan‐4‐one (MPDO) monomer to form homopolymers and/or styrene/MMA copolymers.^[^ ^277^ ^]^ Depending on the conditions used, polymerization can proceed solely through the vinyl propagation or through partial generation of *α*‐ketoester functionalities, resulting in photo‐ and hydrolytically degradable polymers. More nucleophilic 5,6‐benzo‐2‐methyl‐ene‐1,3‐dioxepane (BMDO) was successfully utilized as a comonomer to form degradable polystyrene,^[^ ^278^ ^]^ poly(butyl acrylate),^[^ ^279^ ^]^ and poly(methyl methacrylate)^[^ ^280^ ^]^ copolymers. When BMDO was combined with oligo(ethylene oxide) (OEO)‐based monomers, ATRP allowed to obtain partially degradable, thermoresponsive, and biocompatible terpolymers for potential biorelated applications.^[^ ^281^ ^]^ Copolymerization of 2‐methylene‐4‐phenyl‐1,3‐dioxolane (MPDL) with OEO‐based methacrylic and acrylic esters was also successfully demonstrated.^[^ ^282^ ^]^ Comb‐type polymers of high *M*_W_ (>100 000) were isolated, with MDPL‐level of incorporation being two to three times higher for acrylic than methacrylic copolymers and improved dispersity.

#### 4.10.2 Cleavable Side Chains

Acetals and esters are hydrolytically degradable, while disulfide bonds are redox sensitive. The incorporation of cleavable moieties within bifunctional ATRP initiators^[^ ^283^ ^]^ lead to degradable telechelic polymers, in which every chain can be cleaved into two parts by appropriate stimuli.^[^ ^284^ ^]^ ATRP was used to prepare disulfide‐based injectable triblock copolymer gelators that incorporated 2‐methacryloyloxyethyl phosphorylcholine and NIPAM monomer units.^[^ ^285^ ^]^ Disulfide moieties were reduced to thiols using glutathione under physiological conditions, resulting in concomitant dissolution of micellar gels without generation of side products. Degradable crosslinkers and inimers were utilized for the synthesis of degradable hydrogels and nanogels.^[^ ^286^ ^]^ Moreover, degradable polymers prepared by other polymerization techniques (ring opening, polycondensation) containing suitable sites for ATRP initiation were extended by ATRP.^[^ ^287^ ^]^ Nevertheless, some of these approaches generate materials that degrade into chain fragments with broad molecular weight distributions and/or high MWs. These fragments may still be harmful and more effective degradation is needed.

#### 4.10.3 Star Polymers

**Figure 25:**
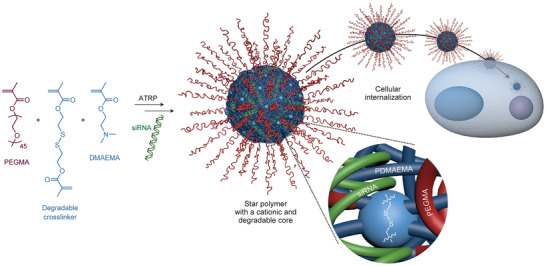
Utilization of disulfide crosslinkers for PEG‐based degradable star polymers prepared via ATRP for the delivery of siRNA. Reproduced with permission.^[^ ^275^ ^]^ Copyright 2015, Springer Nature.

Two strategies were investigated to develop degradable star polymers: i) with multifunctional degradable initiator, or ii) with star cores prepared with a degradable crosslinker to dissociate the arms from star copolymer.^[^ ^288^ ^]^ Using the first approach, four‐arm star polystyrenes were prepared from a multifunctional initiator containing a disulfide and an ester linkage, degraded in two steps under reductive and alkaline environments, respectively.^[^ ^289^ ^]^ Alternatively, the difunctional initiator 2‐hydroxyethyl 2‐ bromoisobutyrate was used to construct core crosslinked star (CCS) polymers by sequential ATRP of styrene, followed by ROP of a hydrolyzable bis‐lactone monomer as a cross‐linking agent.^[^ ^290^ ^]^ Thus, the CCS core was degraded by hydrolysis, while arms contained linear chains. By adjusting the degradable nature of monomers and crosslinkers and by changing the ATRP/ROP sequence, arm‐degradable, partially arm‐degradable and core‐degradable CCSs were formed. One‐pot ATRP copolymerization of (PEG)_45_MA, DMAEMA, and a disulfide dimethacrylate crosslinker yielded biocompatible star polymers with PEG arms and a degradable core via an arm first approach.^[^ ^288a^ ^]^ The star polymers complexed short interfering RNA (siRNA), and the resulting degradable biohybrid system was internalized in for siRNA delivery (**Figure** **25**).

The ability of block copolymers designed by ATRP of acting as nanocarriers was exploited not only in the field of medicine, but also for agriculture. Star polymers with a cyclodextrin core and PAA‐*b*‐poly(N‐isopropylacrylamide) (PNIPAM) block copolymer arms were employed for the controlled release of an antimicrobial agent, due to their pH and temperature responsiveness, which changed with block lengths.^[^ ^291^ ^]^ The star polymers exhibited significant active agent loading capacity, and foliar uptake and translocation in tomato plants, thus showing promise for improving the efficiency and sustainability of agricultural practices.

Another strategy involved the use of biodegradable TA,^[^ ^292^ ^]^ which promoted in vivo esophageal mucoadhesion when combined with PEG.^[^ ^293^ ^]^ TA was also used to form crosslinked conducting polymer hydrogels for spinal cord injury repair.^[^ ^294^ ^]^ The presence of 25 weakly acidic phenolic groups renders TA a potent pH‐responsive material that can be readily functionalized to form multifunctional ATRP macroinitiators. Functionalized TA^[^ ^295^ ^]^ was used as a core to grow PMMA and POEO_300_MA stars by *photo*ATRP. The degradation of star cores was achieved under mild basic conditions. Cytotoxicity tests of both star polymers and their degradation products showed nontoxic character toward various cell lines. Another approach combined TA with Fe^III^ ions and water‐soluble polymers (e.g., polyvinylpyrrolidone (PVP), PEG, poly(dimethyldiallylammonium chloride) (PDDA) and poly(sodium 4‐styrenesulfonate)(PSS)) to generate a series of multifunctional hydrogels.^[^ ^296^ ^]^ Their self‐healing and external stimuli‐responsive character, as well as radical scavenging ability, highlight the potential of RDRP techniques for the synthesis of environmentally benign polymer architectures.

#### 4.10.4 Polymer Brushes

The combination of renewable resources and degradation pathways is an appealing strategy toward sustainable materials. ATRP was applied to form various degradable carbohydrate‐based systems, including chitin, chitosan, dextrane, starch, and cellulose derivatives, the latter showing greater promise from the point of view of degradability.^[^ ^297^ ^]^ Starch is a low‐cost substrate that is amenable to chemical modifications to form materials with better processability and targeted applications. ATRP was used for starch modification through grafting from or grafting onto approaches.^[^ ^298^ ^]^ Generally, starch is functionalized by introducing alkyl bromide initiating sites and subsequently growing polymer chains. Polyacrylamide‐*graft*‐carboxymethyl starch (PAA‐*graft*‐CMS) and poly(hydroxyethyl acrylate)‐*graft*‐CMS (PHEA‐*graft*‐CMS) systems prepared by ATRP were used for in vitro drug release of cephalexin antibiotic.^[^ ^299^ ^]^ Compared with the FRP‐prepared analogs, ATRP‐prepared starch copolymers had longer times of drug release, forming good candidates for drug‐delivery systems.

Cellulose‐based polymers are inherently biodegradable due to their carbohydrate core and their degradability tuned via further modifications.^[^ ^300^ ^]^ However, the decomposition of such systems was a subject of relatively limited studies.^[^ ^301^ ^]^ Styrene‐grafted‐cellulose nanocrystals, with appropriately positioned *o*‐nitrobenzyl ester moiety were subjected to photocleavage of the polystyrene chains, with about 50% efficiency after 5 h of UV irradiation (365 nm).^[^ ^301b^ ^]^ Partial degradation of the cellulose core was also observed and exploited to form *o*‐nitrobenzyl self‐immolative ATRP initiators to build photoresponsive polymers.^[^ ^302^ ^]^ ATRP was used to prepare cellulose‐based polymer brushes with tunable degradability, depending on the polymers grafting density.^[^ ^301c^ ^]^ The brushes decomposition could be achieved either via cleavage of the side chains or degradation of the cellulose backbone. PBA and PDMAEMA brushes grafted from cellulose nanofibers by SI‐ATRP caused the degradation of the backbone for the high grafting density, likely due to the excessive strain imposed by the side chains on the cellulose domains.^[^ ^303^ ^]^ These results imply that the ability of controlling the grafting density of polymer brushes with a cellulose backbone could constitute an effective way to prepare degradable renewable polymers.

#### 4.10.5 Depolymerization Processes

Radical polymerizations are exothermic and exoentropic processes, therefore, depolymerization is possible below the equilibrium monomer concentration [M]_eq_ and above *T*_c_.^[^ ^304^ ^]^ These parameters are strongly affected by the sterics of the *α*‐substituent in the vinyl moiety: e.g., for MMA *T*_c_ ≈ 200 °C, while for methyl *α*‐ethylacrylate (MEA) *T*_c_ = 82 °C, and it is even lower for longer *α*‐alkyl substituent.^[^ ^305^ ^]^ These values are defined by thermodynamic properties, however the depolymerization rate depends on the particular reaction mechanism and can be catalyzed. The depolymerization of chloride‐end capped PMMA was triggered by the reversible activation of the polymer end group in the presence of a Ru‐based catalyst.^[^ ^306^ ^]^ Removal of the formed monomer allowed for 8% recovery of MMA, provided careful control over the temperature (60–120 °C) was maintained to minimize side reactions and ensure the depolymerization proceeding via an iterative “unzipping” mechanism.

**Figure 26:**
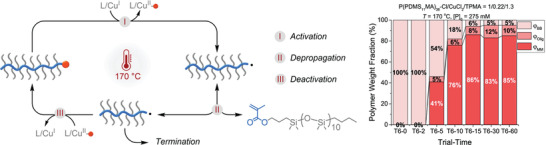
Proposed mechanism of PDMSMA‐Cl macromonomer depolymerization via ATRP catalyzed by Cu/TPMA at 170 °C, and corresponding macromonomer (MM) recovery over time. Reproduced with permission.^[^ ^308^ ^]^ Copyright 2021, American Chemical Society.

A methacrylate‐functionalized polyhedral oligomeric silsesquioxane macromonomer polymerized in a one‐pot reaction to ca. 80% monomer conversion by ATRP at 60 °C, then depolymerized to ca. 60% monomer yield by increasing the temperature to 90 °C.^[^ ^307^ ^]^ A chloride‐capped poly(poly(dimethylsiloxane) methacrylate) macromonomer (PDMSMA‐Cl) was depolymerized at 170 °C, with 80% macromonomer recovery within 10 min (**Figure** **26**).^[^ ^308^ ^]^ The depolymerization occurred via atom transfer between Cl chain ends and the Cu/TPMA catalyst, where the excess TPMA promoted the regeneration of the Cu^I^/L activator at high temperature. Depolymerization yield and selectivity improved with increasing the TPMA loading, while the reaction rate increased with decreasing the macromonomer concentration. This showed that leveraging atom transfer reactions is key to high monomer recovery.

A poly(*n*‐butyl methacrylate) macroinitiator with terminal chlorine chain‐end functionality was depolymerized by ATRP with a copper(II) chloride/tris(2‐pyridylmethyl)amine (CuCl_2_/TPMA) catalyst at 170 °C to recover up to 67% monomer with concurrent distillation on a rotary evaporator. Incubation of the macroinitiators showed evidence of alkyl halide decomposition via lactonization of the chain end, leading to an increase in the thermal stability of the polymer.^[^ ^309^ ^]^ Therefore, further investigation into catalyzed radical depolymerization processes is needed to improve the livingness and monomer yield.

### 4.11 P11: Real‐Time Analysis for Pollution Prevention

Monitoring techniques to follow progress of polymerization in real‐time should be fully integrated with nondestructive and cost‐effective systems. The real‐time monitoring of ATRP provides an additional layer of control over polymer properties and improves the understanding of polymerization mechanisms, besides allowing for limiting the environmental impact of polymerizations.

#### 4.11.1 (Near)Real‐Time ATRP Monitoring

To monitor ATRP in near‐real time, polymerizations were performed in an automated synthesizer connected to an online SEC instrument. The screening time was optimized, and remained limited only by the SEC performance.^[^ ^310^ ^]^ Ultrahigh pressure (UHP) SEC was used to detect star‐star coupling in the synthesis of star polymers via Cu^0^ mediated ATRP.^[^ ^46^ ^]^ UHP‐SEC has high separation resolution compared to conventional SEC instruments, and short‐run times, thus emerging as an efficient monitoring tool, particularly suitable for complex architectures. Automatic continuous online monitoring of polymerization reaction (ACOMP) was applied to ATRP for bulk polymerization of *n*BA.^[^ ^311^ ^]^ ACOMP is based on a continuous extraction, dilution, and conditioning of a small stream pumped from the reactor prior to reaching various analytical instruments.^[^ ^312^ ^]^ For ATRP, this method helped rapid detection of deviations from expected behavior and better understanding of the origin of termination reactions. The concentration of Cu complexes was determined by detecting the signal of X‐Cu^II^/L^[^ ^312^ ^]^ by UV–Vis spectroscopy. The growth of a fluorescent polymer by aqueous ARGET ATRP was monitored in real time via UV–Vis spectroscopy.^[^ ^313^ ^]^ This fluorogenic polymerization was based on copolymerization of an anthracene fluorogenic monomer with PEGMA, which served as a spacer between fluorogenic units to prevent self‐quenching of fluorescence. Fluorogenic ATRP is a promising method for signal amplification and for the development of diagnostic platforms.

**Figure 27:**
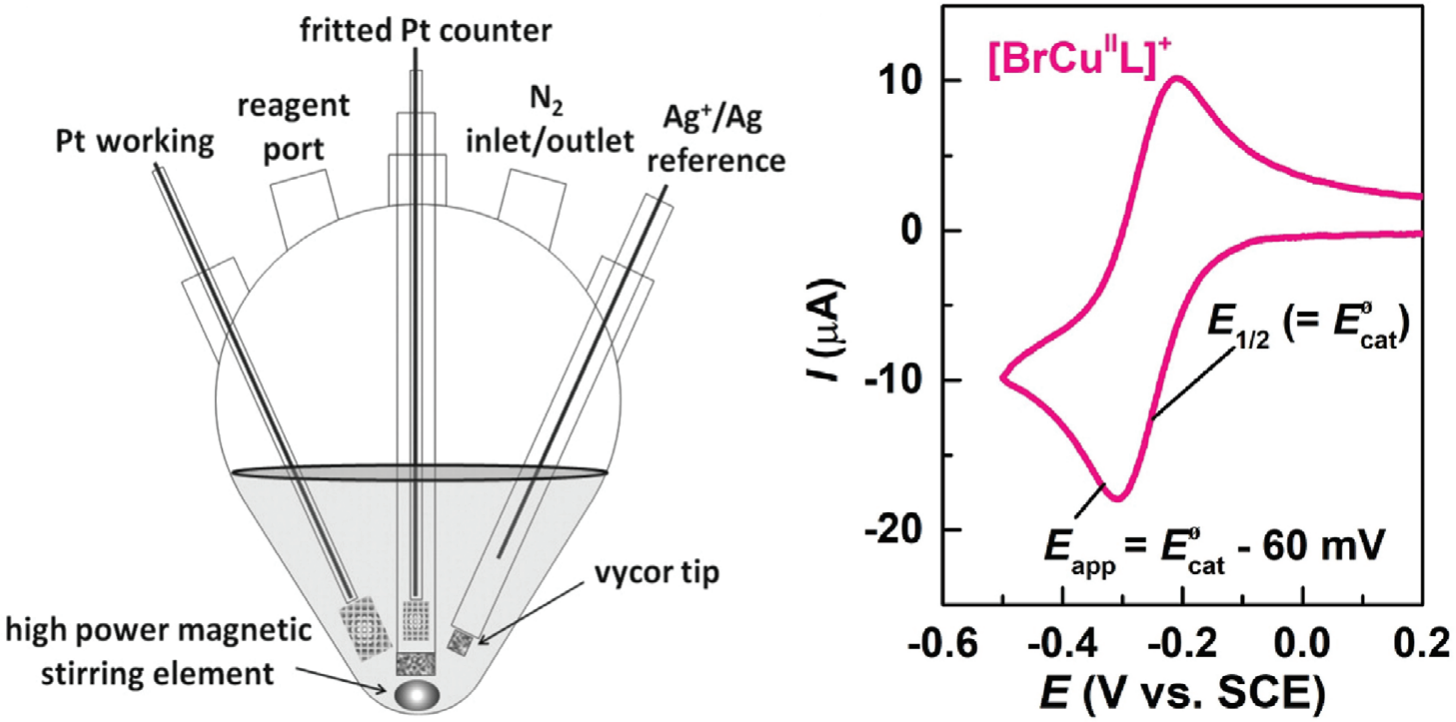
Schematic of a typical electrochemical cell for *e*ATRP (left), and CV of a Cu‐based ATRP catalyst (right). left: Reproduced with permission.^[^ ^25^ ^a]^ Copyright 2011, American Association for the Advancement of Science. right: Reproduced with permission.^[25b]^ Copyright 2019, Elsevier.

External stimuli used for ATRP with activator regeneration can simultaneously serve as polymerization triggers and tools for real‐time monitoring.^[^ ^27^ ^]^ In *e*ATRP, the charge passed during polymerization gives an indication of the extent of termination events (cf. P6: Design for energy efficiency). Moreover, voltametric techniques such as cyclic voltammetry (CV) provide information on the stability of the ATRP catalyst during and after polymerization.^[^ ^25b^ ^]^ The CV response of Cu‐based ATRP catalysts usually consists of a quasi‐reversible peak couple (**Figure** **27**), which should not change significantly during the polymerization, if the catalyst is stable. The CV response could be also modified upon the formation of relatively stable organometallic R‐Cu^II^/L intermediates, formed with very active Cu complexes and radicals in polymerization of acrylates or acrylonitrile.^[^ ^50^ ^]^

A real‐time monitoring of photopolymerizations, including *photo*ATRP, based on light‐coupled optical fibers and NMR spectroscopy was developed.^[^ ^314^ ^]^ An optical fiber, connected to modular LEDs with computer‐controlled light intensity, was centered in conventional 5 mm NMR tubes above the reaction solution. By the continuous recording of NMR spectra, the monomer conversion over time and the increase in the degree of polymerization with conversion could be accurately accessed as the reaction environment was not affected by the measurements. The light was turned on and off to test temporal control over the process.^[^ ^314^ ^,^ ^315^ ^]^ The technique was applied to Cu‐ and Ir‐catalyzed *photo*ATRP, as well as metal‐free ATRP, gaining insights on the effect of different catalytic systems on temporal control.^[^ ^316^ ^]^

#### 4.11.2 Real‐Time Monitoring of SI‐ATRP

**Figure 28:**
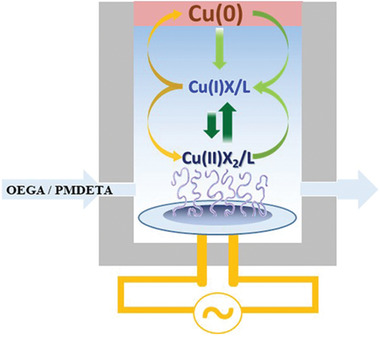
Schematic of a QCM‐D with a functionalized SiO_2_ sensor, to grow brushes via Cu^0^‐SI‐ATRP. Reproduced with permission.^[^ ^164^ ^]^ Copyright 2018, American Chemical Society.

The growth of polymer brushes by ATRP from initiating molecules grafted on a surface was monitored in real‐time through quartz crystal microbalance with dissipation (QCM‐D), surface plasmon resonance (SPR), photonic sensors, AFM, and CV. QCM‐D allows for recording frequency shifts (Δ*f*) corresponding to changes in mass on the surface of a quartz crystal, functionalized with the ATRP initiator, and located in a chamber that is continuously filled with the polymerization solution. The increase in Δ*f* during polymerization enables to calculate the thickness increase of swollen brushes. A frequency–thickness relationship was dependent on the monomer nature, but independent of polymerization rate, initiator, and polymer density.^[^ ^317^ ^]^ This technique was also applied to grow block copolymer brushes.^[^ ^318^ ^]^ SI‐ATRP and QCM‐based real‐time monitoring were coupled to SARA ATRP by using a Cu^0^‐coated quartz slide facing a SiO_2_‐coated sensor functionalized with the ATRP initiator (**Figure** **28**), in the presence of free ligand in the polymerization solution in the chamber. The contributions of supplemental activator and reducing agent pathways were analyzed by quantifying the dissolution of Cu species within a QCM. The control of catalyst diffusion from Cu(0) was exploited to fabricate structured polymer brushes with diverse compositions, when ATRP was performed from surface‐immobilized initiators in the presence of a Cu(0) plate, placed at a determined distance (*d*) from the substrate.

QCM‐based real‐time monitoring of SI‐ATRP was also applied to the growth of various polymer brushes in aqueous solutions with enzymatic degassing, thus eliminating the need for deoxygenation step.^[^ ^319^ ^]^ Better reproducibility in polymerization kinetics was observed in GOx‐assisted ATRPs, compared to conventionally degassed systems, as residual O_2_ traces largely affected the brush growth.

Surface plasmon resonance, SPR, under both angular and fixed angle modes, enables to determine in real‐time the height evolution of polymer brushes. This technique was applied to the ARGET SI‐ATRP of NIPAM on a gold sensor. Above the lower critical solution temperature (LCST) of the monomer (*T*_LCST_ = 32 °C). the brushes collapsed in a rigid layer with three to five lower thickness than at *T* < 32 °C.^[^ ^320^ ^]^ The thermal responsiveness of PNIPAM was exploited for the reversible binding and releasing of proteins, respectively below and above the LCST. Localized SPR (LSPR) was used to monitor the growth of brushes in GOx‐degassed SI‐ATRP.^[^ ^319^ ^]^ Multiplexed arrays of silicon photonic microring resonators were employed for high‐throughput real‐time monitoring of brushes growth.^[^ ^321^ ^]^ Indeed, solutions with different compositions simultaneously flowed across the different regions of a functionalized sensor array. Each step, including the initiator attachment, was tracked and the interfacial properties of the final film were measured in situ. AFM is a powerful analytical tool that can be used for the characterization of polymer brushes, as well as for the fabrication of brush structures across the length scales.^[^ ^322^ ^]^ AFM was used to determine in real‐time the growth kinetics and the morphology of polymer brushes formed by SI‐*e*ATRP on a patterned functionalized gold electrode.^[^ ^323^ ^]^ The instrument consisted of a fluid cell containing a three‐electrode system, installed in the AFM head, which allowed for achieving temporal control over the brushes growth by changing the applied potential. The growth of brushes from an electrode surface could also be followed via CV, by polymerizing a redox‐active monomer.^[^ ^324^ ^]^ As ferrocenyl functional polymer brushes grew on an indium tin oxide (ITO) electrode, the increasing thickness and MW resulted in progressively increasing current intensity in CV. Moreover, the ability of different solvents to swell the brushes affected the reversibility of the CV response.

### 4.12 P12: Inherently Benign Chemistry for Accident Prevention

The last green chemistry principle is sometimes referred to as the “safety principle” and focuses on minimizing potential accidents. Therefore, it is extremely important for both the polymer chemical industry and academic research.^[^ ^56^ ^]^ As the majority of polymerizations are exothermic in nature, the mitigation of dangers associated with the risk of thermal runaway (Trommsdorff‐Norrish effect), is a top priority.^[^ ^42^ ^]^ Therefore, it is fundamental to develop methods to efficiently dissipate the excessive heat generated during the reactions and/or to monitor and instantaneously halt the system on demand. The latter is efficiently achieved in ATRP with activator regeneration, particularly when using external stimuli and highly active catalysts. On the other hand, room temperature polymerizations in aqueous homogenous or aqueous dispersed media (cf. P5: Benign solvent and auxiliaries) help preventing sudden temperature increase.

#### 4.12.1 Halting ATRP on Demand

While ATRP can be generally stopped by exposing the reaction mixture to oxygen, this is impractical for large volume reactors, and immediate stop is complicated by diffusion. *Photo*ATRP and *sono*ATRP are stopped by switching off the light or the sonication, respectively. In *e*ATRP, the applied current or potential must be removed or changed to an oxidizing value in order to convert all the catalyst to the deactivator form. ARGET ATRP can be stopped by introducing an oxidizing agent.^[^ ^325^ ^]^ SARA and Ag^0^ ATRP can be halted by lifting the metallic wire from the solution.^[^ ^326^ ^]^ Effective halting of the process depends on the catalyst activity where more active catalysts have higher *K*_ATRP_, thus lower equilibrium concentration of Cu^I^/L that results in faster polymerization halt when an external stimulus is removed/changed, or an oxidizing agent is added.^[^ ^316^, ^326^ ^]^ For the same reason, polar solvents favor temporal control.

#### 4.12.2 The Ultimate ATRP

**Figure 29:**
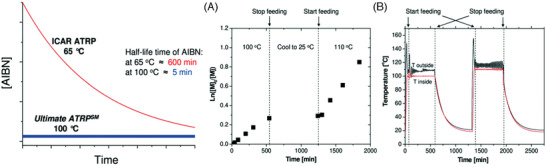
Theoretical concentration of AIBN during the polymerization via ICAR ATRP and in the Ultimate ATRP process. A) Kinetic plot and B) temperature profile during the polymerization of styrene using the Ultimate ATRP process. Conditions: Sty/RX/CuBr_2_/TPMA/AIBN = 1000/1/0.05/0.15/fed; bulk at 100–110 °C; 50 ppm of Cu; feeding rate = 3.33 mL h^−1^ (0.008 equiv. of AIBN versus diethyl 2‐bromo‐2‐methylmalonate initiator in 1 h). Reproduced with permission.^[^ ^327^ ^]^ Copyright 2012, American Chemical Society.

To minimize the potential for accidents, the Ultimate ATRP process^[^ ^327^ ^]^ was developed and commercially applied. This technique is a variant of ICAR ATRP, where the activator regeneration is achieved through slow continuous feeding of a thermal radical initiator to the reaction mixture in a controlled manner, rather than by simple injection of the thermal radical initiator at the beginning of the process. In Ultimate ATRP, precise control of the molar ratio between ATRP activator and deactivator is achieved by tuning the feeding rate, without affecting the livingness of the process or the composition and architecture of the polymer. The concentration of the initiator remains low during the entire feeding time. At the same time, the reaction temperature is sufficiently high to ensure a short half‐lifetime of the radical initiator, in the range of minutes. Therefore, the small amount of instantaneously added initiator is very quickly consumed, thus forming an excess of Cu^II^ deactivator species, and halting the polymerization until more initiator is added to the system, minimizing at the same time the danger of local overheating. The bulk polymerization of styrene via Ultimate ATRP was performed at 110 °C, in the presence of 50 ppm of CuBr_2_ and TPMA ligand, with continuous feeding of a solution of AIBN in toluene at a constant rate of 0.008 equiv h^−1^ (**Figure** **29**).

The reaction was halted after 9 h by stopping the feeding and decreasing temperature. Afterward, the polymerization was restarted at 110 °C, using the same dosage and rate of AIBN solution feeding. Moreover, the conditions were optimized to strongly decrease the reaction time, by decreasing the amounts of solvents, initiator, and catalyst, with real‐time analysis and automation (cf. P11: Real‐time analysis). The viability of this approach was demonstrated for a series of styrene and (meth)acrylates‐derived linear, block and star polymer architectures, and industrially scaled up to 750 gallon capacity.^[^ ^34^ ^]^

#### 4.12.3 Cu Scavengers

**Figure 30:**
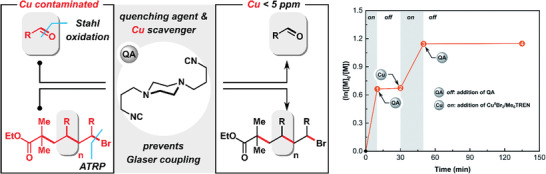
Schematic representation of isocyanide ligands for removal of the copper catalyst and spatiotemporal halting of the ATRP process. Reproduced with permission.^[^ ^67^ ^]^ Copyright 2020, Royal Society of Chemistry.

Another method to halt the ATRP process is to remove the Cu^I^/L species by using an appropriate metal‐ion scavenger. The latter should preferentially bind to Cu^I^ ions, displacing the polydentate amine ligand. As a result, the metallic center can no longer participate in the ATRP equilibrium and the polymerization stops. Therefore, the scavenger can act as a polymerization quenching agent. Commercially available 1,4‐bis(3‐isocyanopropyl)piperazine was utilized to provide temporal control of ATRP through the removal of Cu^I^ species, without compromising the architecture of formed polymers (**Figure** **30**).^[^ ^67^ ^]^ The isocyanide ligand is effectively bound to the copper activator, whereas the addition of a new portion of copper catalyst allowed for the reinitiation of the polymerization reaction.

Importantly, facile purification of the solution was achieved via a syringe filter, and low‐ppm levels of Cu were observed in the final polymers, even under normal ATRP conditions. The isocyanide ligand is nontoxic and has the potential to be utilized as a safety net in strongly exothermic copper‐based processes.

## 5 Summary and Outlook

**Figure 31:**
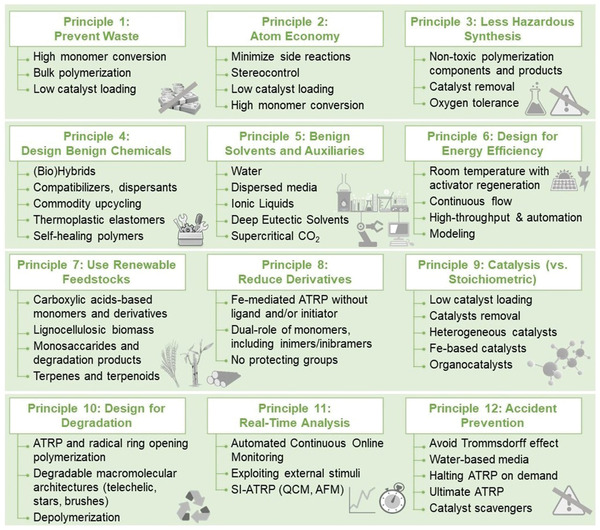
Application of the principles of green chemistry in ATRP.

After 25 years from its invention, ATRP is a technique of choice for the preparation of well‐defined polymers from a broad range of vinyl monomers, and with minimal impact on the environment and human health. In depth mechanistic understanding of ATRP was achieved through systematic evaluation of structure–reactivity relationship of initiators, catalysts, ligands, additives, and monomers, and detailed investigations on the effect of temperature, solvent, and pressure. The development of activator regeneration techniques that use benign reducing agents or external stimuli has largely improved the adherence of ATRP to the twelve principles of green chemistry. These principles are directed toward the design of sustainable chemical processes through the minimization of waste, the atom economy, the reduction of hazards in synthetic processes, the design of safer chemicals, solvents, and auxiliaries, an increase in energy efficiency, the use of renewable raw materials, and rigorous control of the process. ATRP with activator regeneration coupled with highly active and selective catalysts, as well as oxygen‐tolerant or biocompatible systems can be highly energy‐efficient, atom‐economical, ecofriendly, and low risk. Moreover, the precise control over polymer architecture that is achieved in ATRP allows for designing innovative polymers with desired properties and functionalities, derived from nonfossil feedstocks, and amenable to on‐demand degradation or reprocessing and recycling. Therefore, ATRP is poised to be one of the fundamental techniques for the green production of polymers. The most important features of ATRP in its current development status that respond to the twelve green chemistry principles and that have been discussed in this review are summarized in **Figure** **31**.

The necessary transition toward a circular plastic economy and a more sustainable chemical industry requires ATRP and other RDRP techniques to further improve their green character, and expand the biobased, degradable, or reusable nature of their polymeric products. Considering the synthetic aspects, further efforts are needed to enlarge the monomer scope of ATRP in aqueous homogeneous and heterogeneous systems, as well as other green solvents such as DESs. Aqueous media should be increasingly used not only for the synthesis of bioconjugates, but also for innovative bioderived polymers and polymer/inorganic hybrids, to exploit the advantages of rapid ATRP activation/deactivation and diminished terminations. Increasingly more active and particularly more selective catalysts are needed to reduce the polymerization time and energy consumption, minimize the catalyst loading, thus decreasing costs and eliminating purification steps. To this end, it is necessary to develop more effective Fe catalysts and organocatalysts, which remain underexplored in comparison with Cu complexes. Rapid and selective syntheses coupled with oxygen tolerant methods should boost the development of high‐throughput ATRP, which is crucial for fast screening and optimization of catalytic systems, and for biomedical applications. In addition, ATRP modeling should be nurtured, accompanied by machine learning methods to design more effective catalysts and to comprehend structure–property relationships for polymers with complex architecture.

It is imperative to exploit the mechanistic knowledge of ATRP systems to tackle depolymerization processes. Initial studies suggest that atom transfer reactions between conventional ATRP catalysts and alkyl halide chain ends can be used to convert halogen‐capped polymers back to monomers via a controlled radical mechanism. Therefore, efforts should be devoted to maximizing the monomer yield, decrease the extent of side reactions, employ milder conditions, and broaden the scope of functional polymers.

ATRP tolerance to various functional groups promotes the exploration of new monomers derived from renewable sources, and the modification of natural polymers via grafting from or grafting through approaches. While strong focus was put on cellulose modifications, sources such as lignin, monosaccharides, and terpene‐ and terpenoids‐derived monomers need to be explored more extensively as building blocks for green polymers prepared by ATRP, also combined with other techniques. At the same time, the end‐of‐life fate of polymers should be always considered during their creation, which is gaining more attention but is not yet considered mandatory. Thus, on one side, it is important to make polymers even more durable, but on the other side to introduce functionality that can trigger degradation, or use dynamic bonds that simultaneously prolong the material lifetime, promoting its reuse and repurposing, but also degradability.

Overall, ATRP can play a major role toward a sustainable polymer production and circular plastic economy, however technological advancements must be supported by constant dialogue and collaborations with industrial partners. The CRP Consortium at Carnegie Mellon University has successfully promoted the utilization of ATRP in industry and should include companies that target the production of green polymers, focused on sustainable products and practices. In this context, researchers working on ATRP in academy and industry can use a variety of tools, including the twelve principles of green chemistry outlined herein, as well as more quantitative metrics to evaluate the impact of their products and processes. Green chemistry metrics, such as E‐factor, atom economy, process mass intensity, and material and energy efficiencies, could help measure the adherence of ATRP processes to green chemistry principles. Their evaluation allows for designing greener processes, preventing pollution and relying on ecofriendly chemical syntheses. Green chemistry metrics together with life cycle assessment (LCA) and circular economy (CE) indicators give a more holistic sustainability view, starting at the reaction level and then moving to a process level and beyond. LCA quantifies the whole‐of‐life environmental impact, while green chemistry metrics identify hot spots within chemical reaction manufacturing, and CE metrics guide the study of mass flows of resources, indicating potential waste reduction capabilities. These metrics are crucial to transfer products and technologies from laboratories to markets, and to favor the dialogue between researchers and policy makers. Ultimately, the collaboration between universities, industries, and governing bodies will be key to fully exploit the potential of ATRP and other RDRPs toward green polymer production and plastic circularity.

## Conflict of Interest

The authors declare no conflict of interest.
